# Text Mining for Protein Docking

**DOI:** 10.1371/journal.pcbi.1004630

**Published:** 2015-12-09

**Authors:** Varsha D. Badal, Petras J. Kundrotas, Ilya A. Vakser

**Affiliations:** 1 Center for Computational Biology, The University of Kansas, Lawrence, Kansas, United States of America; 2 Department of Molecular Biosciences, The University of Kansas, Lawrence, Kansas, United States of America; Tel Aviv University, ISRAEL

## Abstract

The rapidly growing amount of publicly available information from biomedical research is readily accessible on the Internet, providing a powerful resource for predictive biomolecular modeling. The accumulated data on experimentally determined structures transformed structure prediction of proteins and protein complexes. Instead of exploring the enormous search space, predictive tools can simply proceed to the solution based on similarity to the existing, previously determined structures. A similar major paradigm shift is emerging due to the rapidly expanding amount of information, other than experimentally determined structures, which still can be used as constraints in biomolecular structure prediction. Automated text mining has been widely used in recreating protein interaction networks, as well as in detecting small ligand binding sites on protein structures. Combining and expanding these two well-developed areas of research, we applied the text mining to structural modeling of protein-protein complexes (protein docking). Protein docking can be significantly improved when constraints on the docking mode are available. We developed a procedure that retrieves published abstracts on a specific protein-protein interaction and extracts information relevant to docking. The procedure was assessed on protein complexes from Dockground (http://dockground.compbio.ku.edu). The results show that correct information on binding residues can be extracted for about half of the complexes. The amount of irrelevant information was reduced by conceptual analysis of a subset of the retrieved abstracts, based on the bag-of-words (features) approach. Support Vector Machine models were trained and validated on the subset. The remaining abstracts were filtered by the best-performing models, which decreased the irrelevant information for ~ 25% complexes in the dataset. The extracted constraints were incorporated in the docking protocol and tested on the Dockground unbound benchmark set, significantly increasing the docking success rate.

## Author Summary

Protein interactions are central for many cellular processes. Physical characterization of these interactions is essential for understanding of life processes and applications in biology and medicine. Because of the inherent limitations of experimental techniques and rapid development of computational power and methodology, computer modeling is a tool of choice in many studies. Publicly available information from biomedical research is readily accessible on the Internet, providing a powerful resource for modeling of proteins and protein complexes. A major paradigm shift in modeling of protein complexes is emerging due to the rapidly expanding amount of such information, which can be used as modeling constraints. Text mining has been widely used in recreating networks of protein interactions, as well as in detecting small molecule binding sites on proteins. Combining and expanding these two well-developed areas of research, we applied the text mining to physical modeling of protein complexes (protein docking). Our procedure retrieves published abstracts on a protein-protein interaction and extracts the relevant information. The results show that correct information on binding can be obtained for about half of protein complexes. The extracted constraints were incorporated in a modeling procedure, significantly improving its performance.

*This is a PLOS Computational Biology Methods paper.*

## Introduction

The rapidly growing amount of publicly available information from biomedical research is a modern day phenomena that is likely to continue and accelerate in the future. Most of this information is readily accessible on the Internet, providing a powerful resource for predictive biomolecular modeling. The accumulated data revolutionized structure prediction of proteins in the 80’s [1] and, recently, of protein complexes [2–4] due to the growth of Protein Data Bank (PDB) [5], providing enough structural “templates” for the prediction targets. Instead of painstaking and generally unreliable exploration of the enormous search space, based on the physical “first principles,” nowadays tools can simply proceed to the solution based on similarity to the existing, previously determined structures.

In our opinion, the next stage of this revolution is brewing due to the rapidly expanding amount of information, other than experimentally determined structures, which still can be used as constraints in biomolecular structure prediction [6]. In this paper we present the first, to our knowledge, approach to structural modeling of protein-protein (PP) complexes (protein docking), based on the input from automated text mining (TM) of publications on the Internet.

Protein-protein interactions (PPI) are central for many cellular processes. Structural characterization of PPI is essential for fundamental understanding of life processes and applications in biology and medicine. Because of the inherent limitations of experimental techniques and rapid development of computational power and methodology, protein docking is a tool of choice in many studies. One of the main problems in protein docking [7] is identification of a near-native match among the large, often overwhelming, number of putative matches produced by a global docking scan. To detect the near-native matches at the docking post-processing stage, a scoring procedure is performed by re-ranking of the scan output matches, typically using energy/scoring functions, which are too computationally expensive or impossible/impractical to include in the global search. Such scoring schemes may be based on structural, physicochemical, or evolutionary considerations [8]. For some PPI, information on the docking mode (e.g. one or more residues at the PP interfaces) is available prior to the docking. If this information is certain, there is no need for the docking global scan, and the search can be performed in the sub-space that satisfies the constraints. However, if the probability of such information is <100%, it may rather be included in the post-processing of the global scan, as part of the scoring.

Given the inherent uncertainties of the global-search docking predictions, such independent information on the binding modes is extremely valuable [9]. Such information may be available on the case-by-case basis. However, for docking server predictions that can be used by the broad biological community an automated search for such information can be of great value.

The PPI research is an extremely active field, yielding a vast amount of publications on interacting proteins [10]. These publications quickly become available online (e.g. through PubMed, http://www.ncbi.nlm.nih.gov/pubmed), and are a growing resource for automated mining of the PP binding mode. Many applications (PubMed ENTREZ, NLProt, MedMiner, etc.) utilizing TM techniques have been developed to improve access to the published knowledge [11]. TM converts textual information into database content and complex networks, facilitating development of novel working hypothesis [12]. In biology, TM tools have been used to mine generic or specific information on genes, proteins and their functional relationships. Natural Language Processing (NLP) and Support Vector Machines (SVM) have been used to extract information on connection between proteins in PPI networks [13–19]. Along with the networks of interacting proteins, TM tools have been used to generate a dataset of non-interacting proteins [20]. Full-text articles on metabolic reactions and mutation impacts have been mined by rule-based parser, pattern matching, and entity taggers (protein and gene names along with specific keywords) [21, 22]. Automated TM procedure was developed to predict subcellular localization and function of proteins [23]. TM in combination with the dynamic perturbation analysis has been employed to increase confidence in predicted protein functional site [24], suggesting that if a residue is mentioned in an abstract on the protein structure, it is likely to be in the functional site.

TM approaches are also implemented in many Web-based applications. There are different TM tools for identification of interacting proteins from biological literature and databases [25]. GENIA corpus (a collection of semantically annotated documents) has been specifically designed for testing NLP approaches [26]. PESCADOR extracts a network of interactions from a user-provided set of PubMed abstracts [27]. LAITOR can further filter this mined interactome according to the specific user needs [28]. CRAB extracts data from MEDLINE abstracts, which are relevant to tumor-related chemicals posing risk to human health [29]. PIE utilizes word and syntactic features to effectively capture PPI patterns from biomedical literature [30, 31]. eFIP mines information on phosphorylation and related interactions of a given protein using rule-based NLP [32]. PPInterFinder extracts Medline abstracts on human proteins using co-occurrences of protein names, specific keyword dictionary, and pattern matching [33]. BioQRator can annotate PPI-relevant entity relationships from the biomedical publications [34].

In this paper, we propose the first, to our knowledge, approach to TM constraints for PP docking. Our methodology, by design, is a combination and expansion of two well-developed TM fields: (1) identification of interactors in PPI networks, and (2) detection of protein functional (small ligand) sites. We use the first one as the source of expertise on TM of PPI (existing approaches are concerned with the fact of interaction, not the mode of interaction), and the second one as the source of expertise on TM for structural prediction of the binding sites on proteins (existing approaches are for small non-protein ligands). The method was tested on PubMed abstracts of publications on protein complexes from Dockground (http://dockground.compbio.ku.edu) and showed a significant improvement of the docking success rates.

## Methods

### Text-mining protocol

**Fig 1:**
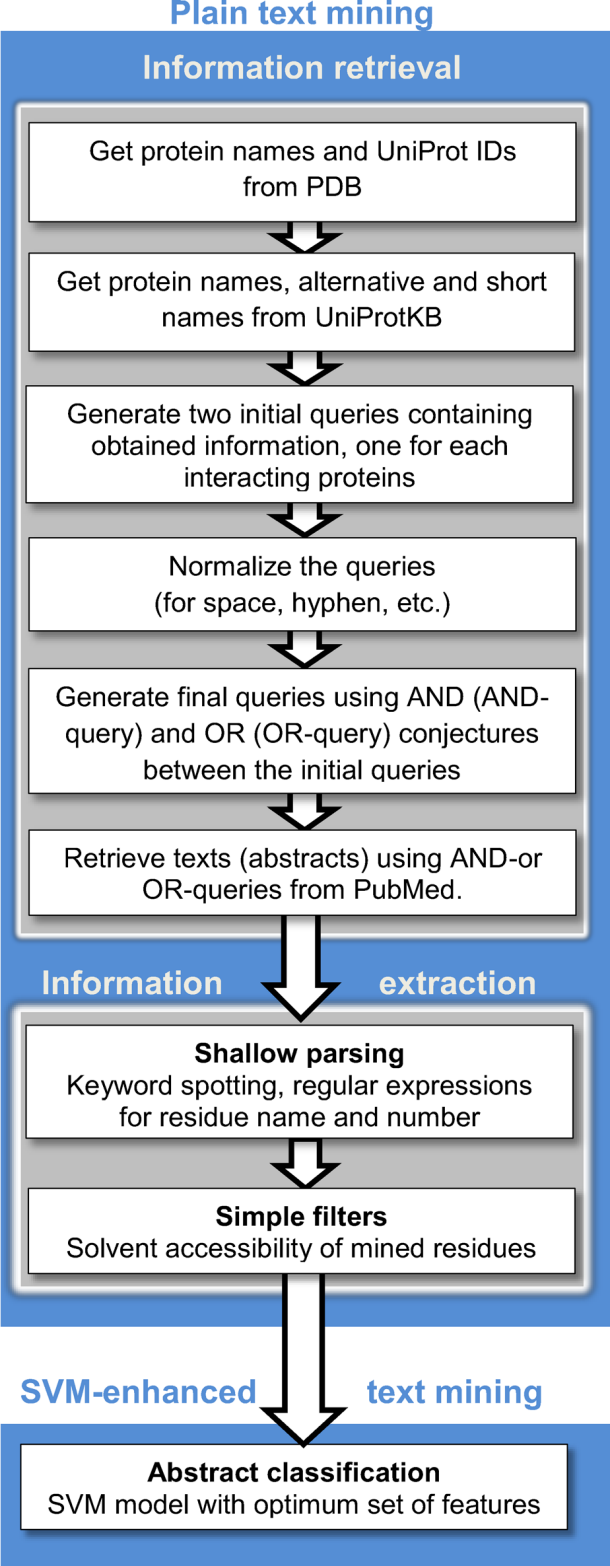
Flowchart of the text mining protocol.

The principal stages of the TM protocol are shown in Fig 1. We divide our procedure into two parts, information retrieval (selecting abstracts containing names of both or either proteins in a complex) and information extraction (detecting occurrence of residues in the retrieved abstracts). The abstracts were further filtered by SVM model with optimal sets of features. The TM tool was benchmarked on 579 PP complexes with known bound X-ray structures from Dockground and applied for re-scoring of the initial docking models for 99 protein pairs from the Dockground unbound benchmark set 3.

### Information retrieval

Protein name and UniProtKB ID, corresponding to the particular PDB code and protein chain, were obtained from PDB. To simulate the “real case scenario” when the structure of the target protein is unknown, PubMed ID (PMID) of the direct citation (publication describing the X-ray structure of the complex) was extracted from the PDB and the publication excluded from the further consideration. To further test our methodology, we also restricted the analysis to abstracts published prior to the direct citation paper. Using the UniProtKB ID, protein information in XML format was acquired from the UniProtKB [35]. Information from both PDB and UniProtKB was accessed through REST (REpresentational State Transfer) Web Services (http://www.rcsb.org/pdb/software/rest.do), (http://www.uniprot.org/help/programmatic_access). For the query construction, at the current stage, we used only recommended, short and alternative protein names, ignoring organism name, classification of the monoclonal antibodies (“CD_antigen” tag), all parts of gene information (name, synonyms, ordered locus names, and open reading frame), E.C (Enzyme Commission) numbers, as well as UniProtKB terms “Uncharacterized protein.” Inclusion of all this additional information into the search queries requires implementation of deep parsers, which is in our plans for the future research.

Protein names were normalized by replacing reserved characters with their URL encodings (spaces replaced by %20, etc.), by removing extra (trailing) spaces, and by hyphen replacement. The query for a protein with a hyphen in its name contains OR-connected versions of the name with hyphen, hyphen removed and replaced by space. For example, query for “IL-15R-alpha” (PDB: 2z3q, chain B) also includes following variations: “IL-15Ralpha”, “IL15R-alpha”, “IL15Ralpha”, “IL%2015Ralpha”, “IL15R%20alpha”, and “IL%2015R%20alpha”. Short names with < 3 symbols were ignored and additional AND-connected keyword “protein” was added to the 3-symbols names.

For 13 PP complexes in the set, protein names coincided with generic, frequently used words (“act” for 1yrt, chain A, UniProtKB: P0DKX7 or “hot” for 2ido, chain B, UniProtKB: Q71T70). To reduce noise, search queries for such complexes contained also MESH terms [36] (combinations of the MESH terms under heading “Biochemical Phenomena”).

For 87 proteins, UniProtKB had section “Cleaved into the following X chains” referring to different domains. In such cases, we considered several scenarios. In the case of exact match between PDB and UniProtKB recommended protein names (29 proteins), we assumed that the PDB structure comprised all the domains mentioned in UniProtKB and included into the query OR-connected recommended name for the entire protein and the names of all the domains. For example, X-ray structure for cationic trypsin (PDB 2xtt, chain B, UniProtKB: P00760) contains both domains (Alpha-trypsin chain 1 and Alpha-trypsin chain 2) mentioned in UniProtKB and the PDB name matches exactly the UniProtKB recommended name. If PDB name matched exactly only one of the domain names (17 proteins), than the query included the name of only this domain along with the recommended protein name. For example, “Protease inhibitor SGPI-1” (PDB 2xtt, chain A, UniProtKB: O46162) is the PDB protein name, which matches exactly one of the cleaved components and does not match recommended UniProtKB name “Serine protease inhibitor I/II”. If the PDB protein name did not match exactly neither UniProtKB recommended name, nor any domain names (41 proteins), we considered only the recommended and the PDB names. For example, “epithelial-cadherin” is the PDB protein name (PDB 2omz, chain B, UniProtKB: P12830), which is not the same as the recommended UniProtKB name “Cadherin-1” and none of the cleaved components (“E-Cad/CTF1”, “E-Cad/CTF2”, “E-Cad/CTF3”). String comparison was performed using Perl module Text::Levenshtein, which implements Levenshtein similarity string matching algorithm (http://search.cpan.org/dist/Text-Levenshtein/lib/Text/Levenshtein.pm).

After constructing two queries, “query1” and “query2”, one for each protein in a particular complex, two final queries were assembled: “query1 AND query2*”* (termed here as AND-query) and “query1 OR query2*”* (OR-query). The AND- and OR-queries were submitted to ESearch and EFetch modules of NCBI EUtilies tool (http://www.ncbi.nlm.nih.gov/books/NBK25501). To keep track on which protein is studied in the retrieved abstracts, two parts of the OR-query were submitted separately. Maximum of 100,000 PubMed abstracts with publication dates between January 1, 1971 and November 30, 2014 were retrieved for each submitted query.

### Information extraction

Abstracts of publications corresponding to 579 complexes, retrieved by the E-utilities from the PubMed (the number of abstracts varies for different types of queries, see Results and Discussion), were searched for the residues using regular expressions (Table 1) obtained by the manual inspection of 100 abstracts that mention residues. We considered patterns with only three-letter or full residue names, since mining of one-letter residue abbreviations requires deep parsing of the surrounding text, which is beyond the scope of our current study. However, if keywords related to mutagenesis studies (“mutation”, “mutagenesis”, “mutagen”, “mutant”, “substitution”) were spotted, one-letter abbreviations for mutation (e.g., “S4A”) were included in the search patterns. For the mutations, both original and substitution residues were taken (e.g., for the pattern “S4A”, both Serine 4 and Alanine 4 were considered as the mined residues).

**Table 1:** Regular expressions for amino acids in the information extraction part of the text mining protocol.

Parameter	Value
**Number**	[1–9][0–9] ^a^
**Amino acid (AA)**	[Ala,…, Val] OR [ala,…, val] OR [ALA,…, VAL] ^b^
**Three letter residue**	AA(no space)Number OR AA(space)Number OR AA–Number **|** AA(Number)
**Full AA**	[Alanine,…,Valine] OR [alanine,…,valine] ^c^
**Full word residue**	Full_AA(no space)Number OR **|** Full_AA(space)Number OR Full**_**AA–Number OR Full_AA(Number)
**Single AA**	[A,….,V] ^d^
**Single letter mutation**	Single_AA(no space)Number(no space)Single_AA
**Three letter mutation**	AA(no space)Number(no space)AA OR AA-Number(no space)AA

^a^ Non-zero digit followed by any number of digits

^b^ Three-letter abbreviation for standard amino acids

^c^ Full name of amino acid

^d^ One-letter abbreviation for amino acid

Since residues participating in docking are on the protein surface, the names and numbers of the extracted residues were checked against the names and numbers of the surface residues from the original PDB file. For the AND-query, the check was performed against both chains of the original complex, whereas for the OR-query, the examination was done only for the protein mentioned in the retrieved abstract (to reduce noise due to the accidental match of the residue name and numbers). Surface residues were defined as those with ≥ 25% of their surface exposed to solvent [37]. The solvent accessible area was calculated by the program surfv [38]. Only the residues with both name and number matching the residues from the original PDB file were considered further (we termed them "identified residues"). In the case of mismatch between PDB and UniProt sequence numbering, we mapped the UniProt sequence on the PDB one as in Ref. [39]. An identified residue was considered correct if any of its heavy atoms was ≤ 6 Å from any heavy atom of the interacting protein in the co-crystallized complex, which means that the residue is at the PP interface. Performance of the TM protocol for a particular PPI, for which a query extracted *N* abstracts containing residues, was quantified as a fraction of correct (interface) residues among all identified residues

$$P_{\text{TM}}=\frac{\sum_{i=1}^{N}N_{i}^{\mathrm{int}}}{\sum_{i=1}^{N}\left(N_{i}^{\mathrm{int}}+N_{i}^{\text{non}}\right)},$$ (1)

where $N_{i}^{\mathrm{int}}$ and $N_{i}^{\text{non}}$ are numbers of interface (correct) and non-interface (incorrect) residues in abstract *i*.

### Generation of feature sets for SVM models

We generated a set of features by handpicking 60 words from carefully read randomly selected 21 PPI abstracts and 43 non-PPI abstracts (Table 2). Subsets of 50, 40, 30, 20 and 10 features were also selected based on our understanding of importance of a feature for PPI description. We refer to these sets of features as *manually selected*, abbreviated as MF*xx*, where *xx* is the number of features in the set.

**Table 2:** Sets of features (stems) for SVM models. Manually selected features are sorted alphabetically and automatically selected features are sorted based on the ratio δ (Eq 5) large to small. PPI-relevant features are in bold.

Number of words in a bag	Bag of words
***Manual selection***	
**60**	activ, **affin,** alloster, **associ, attach, between, bind, bond, bound**, **catalyt**, **chang**, cleavag, cofactor, **complex, conform**, cooper, conjug, **conserv**, **contact,** cycliz, delet, diminish, **direct, domain**, downstream, enhanc**, enzym**, facilit, growth, increas, **induc**, induct, inhibit**, interact, interfac**, involv, **linkag,** mechan, metabol", modifi, modul, phosphoryl, **positio, preferenti, proxim,** reassoci**, receptor,** recognit, redox, regulatori, **signal, specif, stabil**, stimul**, substrat**, suppress, **surfac**, **target, transform, trigger**
**50**	affin, alloster, associ, attach, bind, bond, bound, catalyt, chang, cleavag, complex, conform, conserv, cooper, contact, cycliz, delet, diminish, direct, domain, downstream, enhanc, enzym, facilit, growth, increas, induc, inhibit, interact, interfac, involv, linkag, mechan, metabol, modifi, modul, preferenti, reassoci, recognit, regulatori, signal, specif, stabil, stimul, substrat, suppress, surfac, target, transform, trigger.
**40**	affin, alloster, associ, attach, bind, bond, bound, catalyt, cleavag, complex, conform, conserv, cooper, contact, cycliz, delet, diminish, domain, enhanc, enzym, facilit, increas, induc, inhibit, interact, interfac, linkag, mechan, modifi, modul, preferenti, recognit, regulatori, specif, stabil, substrat, surfac, target, transform, trigger.
**30**	affin, alloster, associ, attach, bind, bond, bound, cleavag, complex, conform, conserv, contact, cooper, domain, induc, interfac, interact, linkag, mechan, modifi, modul, preferenti, recognit, regulatori specif, stabil, surface, substrat, target, transform
**20**	alloster, bind, bond, bound, cleavag, complex, conform, contact, conserv, domain, induc, interfac, interact, mechan, modul, preferenti, recognit, specif, stabi, surface.
**10**	alloster, bind, complex, conform, conserv, contact, induc, interface, interact, recognit.
***Automated selection***	
**143**	polymorph, **interfac**, **energi, bond**, **free**, antibodi, **beta**, phenotyp, patient, promot, light, **degre**, **conjug**, gene, **affin**, **label,** diseas, filament, affect, **signal, ca2+, crystal,** properti, **complex, interact,** resist, level, **valu,** detect, membran, contain, **contribut**, inclu, **terminu**, **produc,** genera, regul, shown, examin, transcript, normal, lower, **time**, **base**, **stabil**, express, **critic**, phosphoryl, **subunit**, function, assai, **peptid, catalyt, surfac,** enhanc, investig, **format, positio**, **determin, residu**, **bind, loop,** rate, **effici**, report, factor**, molecul, inhibit**, **prolifer, deriv,** sequenc, alter, singl, mediat, **structur, purifi,** induc, depend, dna, **reveal,** sensit, **receptor**, compar, **model**, **cleavag, via, product**, **target, variant, specif**, loss, **growth**, potenti, **requir,** essenti, caus, **decreas**, low, **substrat,** associ, mechan, **conform**, **fold**, **contrast**, similar, type, **involv**, found, **novel**, region, **exhibit,** wild, vitro, **observ, develop**, **fragment,** famil**, conserv,** cell, **identifi,** stud, reduc, **provid,** demonstr**, acid, data, link,** effect, presenc, activ, **result**, role, domain, **chain**, **enzym, form, alpha, index**, site, increas, suggest, **mutant**, protein

We also generated a set of features by automated counting of words in the abstracts. We refer to this set and all of its subsets as *automatically selected*, abbreviate as AF*xx*, where *xx* is the number of features in the set. For this set, *L*_pos_ = 450 positive and *L*_neg_ = 855 negative abstracts, respectively satisfying conditions

$$\begin{array}{l} \left[N_{i}^{\mathrm{int}}-N_{i}^{\text{non}}>4\right]\;\text{OR}\;\left[N_{i}^{\text{non}}=0\;\text{AND}\;N_{i}^{\mathrm{int}}>0\right]\;\text{and}\\ \left[N_{i}^{\text{non}}-N_{i}^{\text{int}}>4\right]\;\text{OR}\;\left[N_{i}^{\text{non}}>0\;\text{AND}\;N_{i}^{\mathrm{int}}=0\right], \end{array}$$ (2)

were selected from 1,523 abstracts retrieved by the AND-queries. Positive and negative abstracts were further randomly split into training (80% of abstracts, or $L_{\text{pos}}^{\text{train}}$ = 360 positive and $L_{\text{neg}}^{\text{train}}$ = 684 negative abstracts) and validation (remaining 20%) sets, and features were selected from the training set.

Specific protein and amino acid names were excluded from the counting, as they were part of the queries in the TM protocol. Stop words (“and”, “as”, “because”, “the”) were also purged from the abstracts. The abstracts were subjected to the tokenizer [40] for the suffix stripping by the Porters stemming algorithm [41] in order to get the stem (root) forms of the remaining abstract words. We slightly modified the original algorithm so that a root of a word would accommodate wider variability in the spelling of words with the same meaning. For example, words “include” and “inclusion” are counted by the root “inclu-”, words “mutant”, “mutagenesis”, “mutation”, “mutagen”, “mutated”, “mutations”, “mutation” are accounted for by the root “muta-”, etc.

The normalized counts for each stem (feature) were calculated separately for the positive (*k* = pos) and the negative (*k* = neg) abstracts in the training set

$$f_{k}\left(m\right)=\frac{1}{L_{k}^{\text{train}}}\sum_{i=1}^{L_{k}^{\text{train}}}J_{i}\left(m\right),$$ (3)

where *J*_*i*_(*m*) is the number of times feature *m* appears in abstract *i*. All features satisfying conditions

$$\left|f_{\text{pos}}\left(m\right)-f_{\text{neg}}\left(m\right)\right|>0.02\;\text{and}\;f_{\text{pos}}\left(m\right)+f_{\text{neg}}\left(m\right)>0.2$$ (4)

were selected for the full set AF143 (automatically selected 143 features). The criteria are meant to balance a maximal number of features and a strong signal. The full set was sorted based on the ratio

$$\delta \left(m\right)=\frac{f_{\text{pos}}\left(m\right)-f_{\text{neg}}\left(m\right)}{f_{\text{pos}}\left(m\right)+f_{\text{neg}}\left(m\right)}$$ (5)

and consists of 76 PPI-relevant (*δ*(*m*) > 0) and 67 PPI-non-relevant (*δ*(*m*) < 0) features (Table 2).

### SVM models

We define an SVM model as an SVM classifier with a kernel function, trained and validated with a particular set of features. For the training and validation of the SVM models we used readily available SVMLight [42, 43] with polynomial, *K*(*X*_*i*_,*X*_*j*_) = (*αX*_*i*_ *X*_*j*_ + *C*)^*d*^, and radial-base (RBF), *K*(*X*_*i*_,*X*_*j*_) = exp(−*γ*|*X*_*i*_ − *X*_*j*_|^2^) kernel functions (*X*_*i*_ and *X*_*j*_ are support and test feature vectors). We tested different values of parameters *d* and *γ* while parameters *α* and *C* had default values (*α* = 1 and *C* = 0), and distinguished a particular case of the polynomial kernel with *d* = 1, as the linear kernel. We have also investigated how results are affected by varying degree *d* of polynomial and parameter *γ* of RBF kernels. Validation of the SVM models was carried out in the classification mode where an abstract was identified as positive or negative depending on the sign of the SVM-score. In some cases, abstracts with SVM scores close to zero (within a margin) were considered as “unclassified” and excluded from the performance evaluation.

All SVM models were trained on 1044 abstracts and validated on different 261 abstracts (see above). Performance of an SVM model was evaluated in usual terms of precision *P*, recall *R*, accuracy *A* [44], and Matthews’ correlation coefficient *MCC* [45]

$$\begin{array}{l} \begin{array}{l} P=\frac{\text{TP}}{\text{TP}+\text{FP}},\;R=\frac{\text{TP}}{\text{TP}+\text{FN}},\;A=\frac{\text{TP}+\text{TN}}{\text{TP}+\text{FN}+\text{TN}+\text{FP}}\;\text{and} \end{array}\\ MCC=\frac{\text{TP}\times \text{TN}+\text{FP}\times F\text{N}}{\sqrt{\left(\text{TP}+\text{FP}\right)\left(\text{TP}+\text{FN}\right)\left(\text{TN}+\text{FP}\right)\left(\text{TN}+\text{FN}\right)}}, \end{array}$$ (6)

where TP, FP, TN, and FN are, correspondingly, the numbers of correctly identified positive, incorrectly identified positive, correctly identified negative and incorrectly identified negative abstracts in the validation set.

### Docking with text-mining constraints

Basic TM protocol with the OR-queries was used to mine residues for 99 complexes from the Dockground benchmark set 3 [46], containing the unbound X-ray structures for the co-crystallized complexes (bound structures). Queries for individual proteins and 63 binary complexes were generated as described above. For 36 multimeric complexes, queries were generated using OR-combinations of queries for all monomers in a multimeric chain (e.g., for complex AB: CDE the OR-query was “(queryA OR queryB) OR (queryC OR queryD OR queryE)”). Abstract of publications on the X-ray structure of the co-crystallized complex were excluded from consideration using corresponding PMID from the PDB entry. For validation, the extracted residues were matched to the residues in the bound structures of the dataset (numbering and chain IDs in the bound and the unbound structures is often different). Extracted residues were ranked, in descending order, separately for each interactor (single or multimeric) by the confidence function

$$f(R)=\min\left(10,\;\sum_{i=1}^{N_{R}}a_{i}\right),$$ (7)

where *N*_*R*_ is the total number of distinct abstracts, in which residue *R* is mentioned, and *a*_*i*_ = 2, if abstract *i* was retrieved by the AND-query and *a*_*i*_ = 1, if the abstract was retrieved by the OR-query only. Top five residues for each interactor were used as constraints in our GRAMM docking program [47] giving an extra weight (proportional to *f*(*R*)) to the scoring function if the identified residue was at the interface of a docking model. The upper limit of 10 in Eq 7 was chosen to balance the diversity of low confidence (*f* = 1) vs. high confidence (*f* = 10) constraints and potential overrepresentation of a residue in publications (very high *f* values). If > 5 residues had the highest *f* values, then preference was given to the residues with scores containing more contributions from the abstracts retrieved by the AND-queries. Otherwise, the excess residues were removed from the list randomly.

For validation, the residues at the crystallographically determined interface (reference residues) were extracted from the co-crystallized complexes using 6 Å distance cutoff between the heavy atoms of the proteins in the complex. All pairs of these interface residues were ranked in ascending order by the distance between their C^α^ atoms. The top three pairs were submitted to GRAMM with the highest possible confidence score 10 (reference constraints).

The unbound structures were docked by GRAMM once using the TM constraints and then, for comparison, the reference constraints. The output of the global low-resolution docking scan consisted of 20,000 matches, with no post-processing (except for the removal of redundant matches). These matches were subjected to scoring by the sum of the *f* values (Eq 7) if constraints were generated for the complex. If no constraints were generated, the score was zero. All matches were then re-sorted according to these scores. The quality of a match was assessed by C^α^ ligand interface root-mean-square deviation, *i*-RMSD (ligand and receptor are the smaller and the larger proteins in the complex, respectively), calculated between the interface of the docked unbound ligand and corresponding atoms of the unbound ligand superimposed on the co-crystallized bound structure.

## Results and Discussions

### Basic text mining

#### Overall performance of two query types

The ultimate success of our TM approach relies heavily on the text pool obtained during information retrieval stage (Fig 1). Queries for mining texts on interactions of two proteins often are generated based on the co-occurrence principle [28], requiring that information on *both* proteins be presented in the abstract of a publication (AND-query, see Methods). For generating docking constraints, however, it could be desirable to extract a more diverse text set by requiring presence of information on *either* protein (OR-query). This could be especially helpful for proteins that bind several partners at the same interface. However, the “brute-force” use of the OR-queries may also result in many irrelevant abstracts (allosteric sites, substrate preference, signaling and conformational changes, etc.).

To clarify this issue, we have analyzed abstracts for 579 protein complexes from Dockground retrieved by the AND- and OR-queries. The original publications, describing the PDB structures of the complex, were excluded from consideration. The AND-queries retrieved 220,603 abstracts for 277 complexes, and 18,670 residues (with the names that match features in Table 2) were extracted from 11,732 abstracts for 193 complexes. The application of the simple filters (see Methods) reduced these numbers to 1,375 residues (identified residues) in 1,660 abstracts for 128 complexes. Of those, 571 residues for 108 complexes were found to be correct (at the PP interfaces). For 21 complexes, all identified residues were correct (*P*_TM_ = 1), and for 20 complexes, all identified residues were outside the interface (*P*_TM_ = 0). The OR-queries retrieved 2,640,816 abstracts for 492 complexes; and 207,931 residues were extracted from 150,060 abstracts for 431 complexes. Residue filtering resulted in 5,781 identified residues in 18,528 abstracts for 328 complexes, out of which 1,919 residues in 273 complexes were correct. All identified residues were correct in 36 complexes, and no interface residues were identified for 55 complexes. All abstracts retrieved by the AND-queries were retrieved by the OR-queries as well.

**Fig 2:**
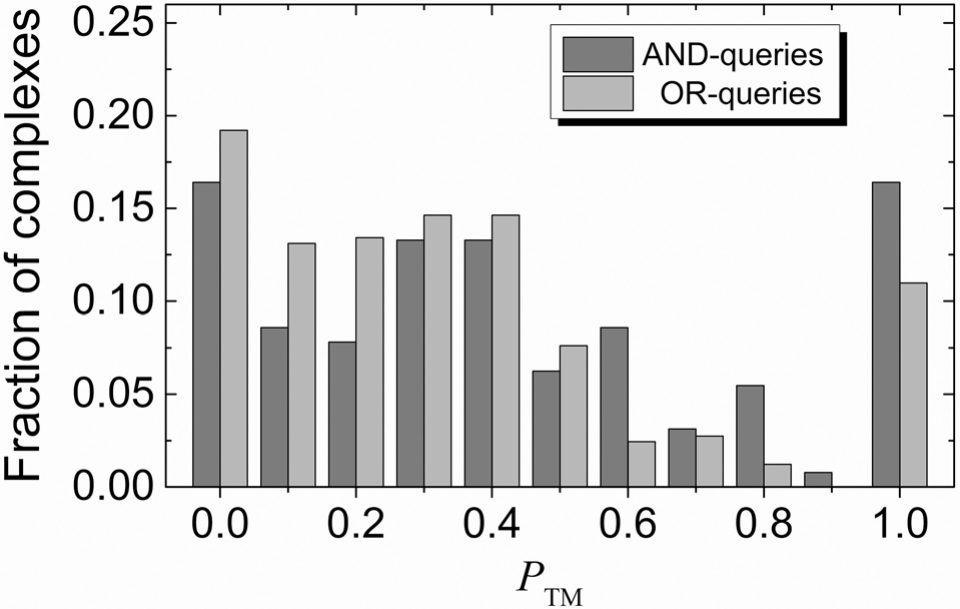
Distribution of complexes according to the quality of the basic TM. The TM performance is according to *P*_TM_ (Eq 1). The distribution is normalized to the total number of complexes for which residues were identified (column 3 in Table 3).

**Fig 3:**
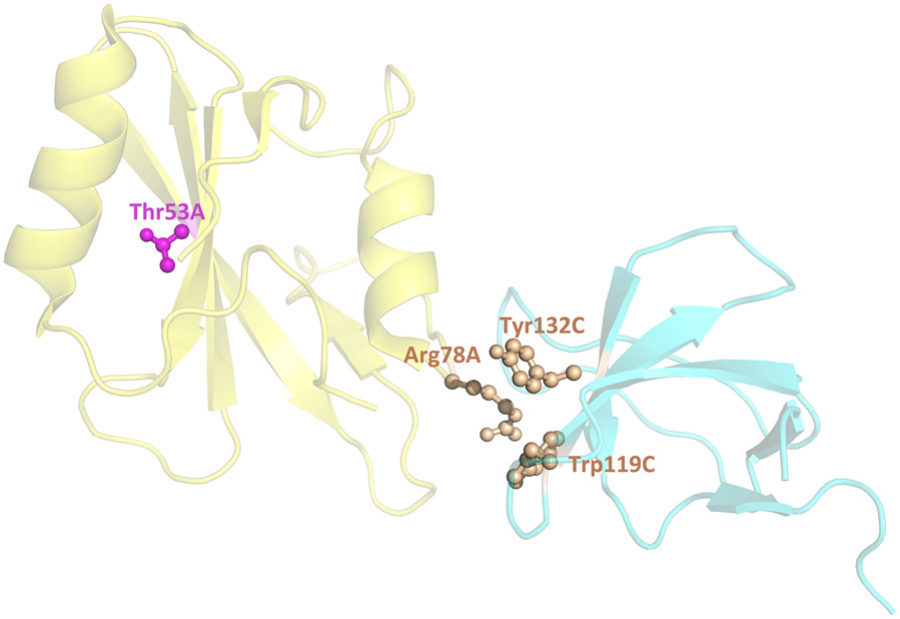
Examples of residues extracted from an abstracts retrieved by OR-query. The structure, chain ID, and residue numbers are from 1m27. Interface and non-interface residues are in brown and magenta, correspondingly.

Comparison of the overall basic TM performance for AND- and OR-queries (first two data rows in Table 3) suggests significantly higher coverage, with comparable accuracy for the OR–queries. However, as data in Fig 2 indicates, the OR-queries also extracted many irrelevant abstracts with non-interface residues (bars for the OR-queries with weaker TM performance [smaller *P*_TM_ values] are larger than the corresponding bars for the AND-queries). For example, for SH2D1A-p59Fyn complex (1m27) AND-query did not retrieve any abstracts, whereas OR-query retrieved 6 abstracts, from which 3 interface and 1 non-interface residues were extracted (Fig 3; for the detailed description, see S1 Text). S1 Fig and S1 Text provide more examples of the basic TM performance with different *P*_TM_ and a detailed explanation of what residues were extracted from the abstracts, retrieved by the AND- and OR-queries.

**Table 3:** Performance of basic and SVM-enhanced TM protocols. The SVM models were trained and tested on abstracts retrieved by the AND-queries. Best models were applied to abstracts retrieved by the OR-queries (see Methods). Total number of complexes in the dataset is 579, if not specified otherwise.

Query type	SVM model	*L*_tot_ ^a^	*L*_int_ ^b^	Coverage (%) ^c^	Success (%) ^d^	Accuracy (%) ^e^
***Basic text mining (numbering of residues from PDB only)***						
AND		128	108	22.1	18.7	84.4
OR		328	273	56.6	47.2	83.2
***Basic text mining (numbering of residues from PDB and UniProt)***						
AND		142	118	24.5	20.4	83.1
OR		342	283	59.1	48.9	82.7
***Basic text mining (numbering of residues from PDB*, *only abstracts prior original publication)***						
AND		96	75	16.6	13.0	78.1
OR		268	202	46.3	34.9	75.4
***SVM-enhanced text mining (numbering of residues from PDB only)***						
OR	MF50L	266	211	45.9	36.4	79.3
OR	AF138L	269	213	46.5	36.9	79.2
OR	AF24L	253	193	43.7	33.3	76.3
**Basic text mining on benchmark 3 (99 complexes, numbering of residues from PDB only)**						
OR		93	82	93.9	82.8	88.2

^a^ Number of complexes for which TM protocol found at least one abstract with residues

^b^ Number of complexes with at least one interface residue found in abstracts

^c^ Ratio of ***L***_**tot**_ and total number of complexes

^d^ Ratio of ***L***_**int**_ and total number of complexes

^e^ Ratio of ***L***_**int**_ and ***L***_**tot**_

#### Correction for different residue numbering

For 430 out of 1158 monomers, the numbering of residues in PDB files did not match that in the UniProt. For these monomers, we modified the filtering of the initial pool of extracted residues (described above), which resulted in 1,619 identified residues in 2,028 abstracts for 142 complexes, for AND- queries; and 6,735 identified residues in 20,040 abstracts for 342 complexes, for OR- queries. All identified residues were correct for 25 and 31 complexes, and no interface residues were identified for 24 and 59 complexes for AND- and OR-queries, respectively. Analysis of these results (third and fourth data rows in Table 3 and S2 Fig) suggests that the numbering mapping only slightly improved TM performance.

#### Mining of abstracts published before the PDB structure paper

To further test the predictive power of our approach, for each complex, we considered only abstracts with publication date earlier than that of the paper on the PDB structure. This reduced the pool to 84,366 abstracts for 263 complexes, and 1,586,097 abstracts for 487 complexes retrieved by the AND- and OR-queries, respectively. For AND- queries, 7,956 residues were extracted from 3,944 abstracts, and standard residue filtering (see Methods) resulted in 776 identified residues in 814 abstracts for 96 complexes. For OR- queries, 114,472 residues were extracted from 81,418 abstracts, and standard residue filtering resulted in 3,731 identified residues in 9,321 abstracts for 268 complexes. All identified residues were correct for 21 and 29 complexes, and no interface residues were identified for 21 and 66 complexes, for AND- and OR-queries, respectively. The analysis of these TM results (5^th^ and 6^th^ data rows in Table 3 and S3 Fig) showed no significant change in the TM performance.

### SVM-enhanced text mining

#### Optimization of SVM models

For the manual mode of feature selection, we considered full MF60 set and five of its subsets, MF50, MF40, MF30, MF20 and MF10 (upper part of Table 2). For the automated mode of feature selection, we started with the full AF143 set (lower part of Table 2) and gradually remove features with smallest |δ(m)| (Eq 5). For each subset, we trained and tested SVM procedure with several different kernels, with and without a margin for the abstract classification (see Methods).

Introduction of the margin only slightly changes SVM performance (S4 and S6 Figs for the MF*xx* sets with linear and RBF γ = 1 kernels, respectively) and filters out considerable number of the abstracts (e.g., for the MF50 set, 13 and 223 abstracts were classified within the 0.05 margin by the linear and the RBF γ = 1 kernels, respectively). Results for the AF*xx* sets and other kernels do not show significant change in the performance of SVM with the margin as well (examples of data for the linear kernel are compared in S8 and S9 Figs). Varying degree *d* of the polynomial (S5 and S10 Figs) and parameter γ of the RBF (S7 and S11 Figs) kernels for both MF*xx* and AF*xx* sets also did not change the SVM performance significantly. Thus, for simplicity, we present and analyze results only for the linear (polynomial *d* = 1) and RBF *γ* = 1 kernels without the margin. Hereafter, we will abbreviate SVM models as AB, where A stands for the feature set (see Methods) and B = L, R for linear or RBF kernels, correspondingly.

**Fig 4:**
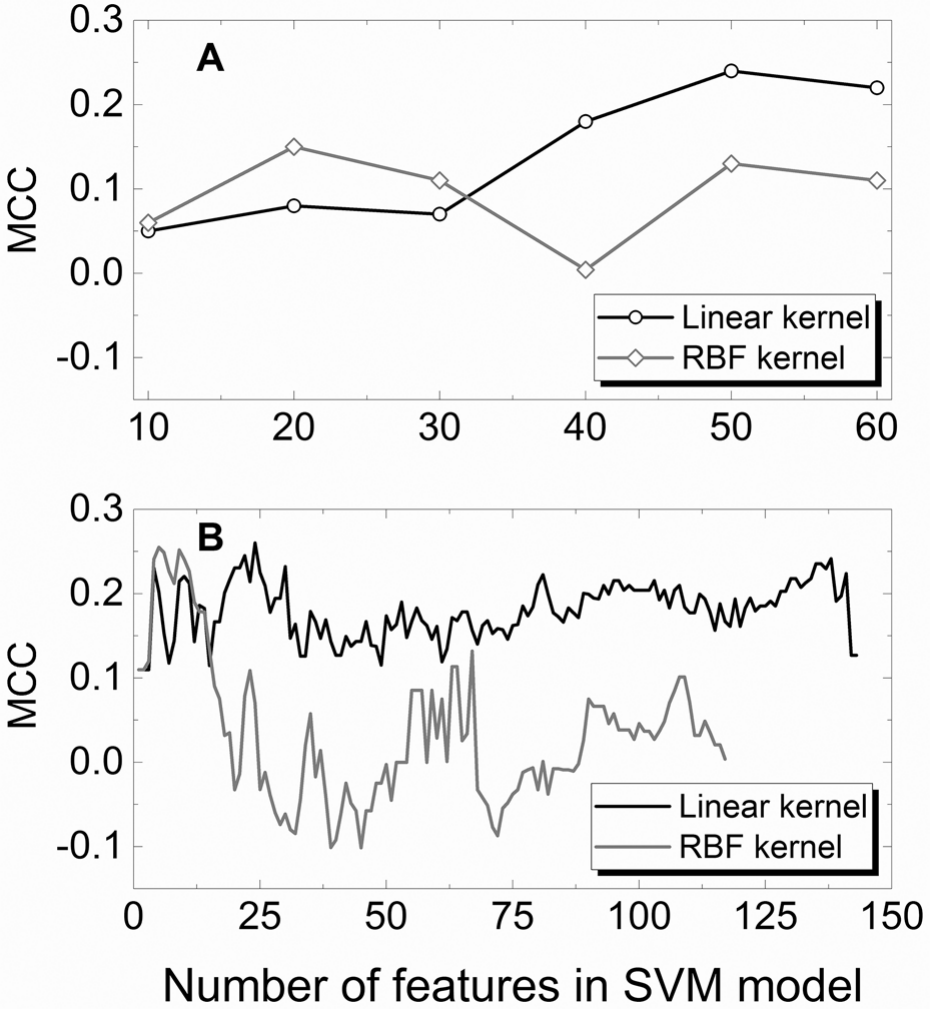
Matthews correlation coefficient vs. number of features in SVM model. The Matthews correlation coefficient (MCC) is calculated according to Eq 6. The features were selected manually (A) and in automated mode (B), for linear and RBF SVM kernels. The data was obtained on the validation set of 261 abstracts. The SVM models were trained on 1,044 abstracts (see Methods).

In this study, we utilized a binary SVM classifier (abstracts are categorized as either positive or negative). Then, the performance of our SVM models can be quantified by a single measure, Matthew correlation coefficient, MCC (Eq 6) and the optimum SVM model would have the maximum MCC value [45]. Results, presented in Fig 4, show that three SVM models (MF50L, AF138L, and AF24L) have approximately the same maximum MCC value ~0.25. The AF138L model has the best recall (57.8%), but worst accuracy and precision (64% and 48.1%, respectively), whereas the AF24L model achieved the best accuracy and precision (66.7% and 51.7%, respectively), but the worst recall (51.1%). The MF50L model has all parameters between the AF138L and AF24L models (Table 4). The variations in the model parameters do not exceed 10% (Table 4). Thus we kept all three models for further consideration.

**Table 4:** Classification of abstracts in the test set by the three optimal SVM models. Total number of abstracts 261 (90 PPI-relevant and 171 non-PPI).

SVM model	TP	FN	TN	FP
MF50L	48	42	123	48
AF138L	52	38	115	56
AF24L	46	44	128	43

No models with the RBF kernel had similar performance, except the AF*xx* sets with *xx* < 15 (Fig 4). Such small number of features in the SVM model is clearly not enough for statistically reliable results and such models were discarded. As number of features in the model increases, the performance of the RBF kernel deteriorates, in particularly, due to the large amount of abstracts with the SVM score close to zero. This correlates with the conclusion of other studies [48–50] that for the most text categorization problems, the best performance is achieved by the linear separation of feature vectors.

Abstract-wise feature selection, irrespective of the number times a feature appears in an abstract, used earlier to extract features for prediction of protein function and localization [23, 51], changes the rank of the initial features. However, the MCC values, calculated for the SVM models with two different methods of feature selections, are in the same range (S12 Fig). Thus significant changes in the SVM performance should not be expected.

#### Performance of SVM-enhanced text-mining protocol

While retrieving abstracts with the residues for significantly larger amount of PPI, the OR-queries bring also up many irrelevant residues. As the first step in mitigating this problem, we filtered 7,991 abstracts for 328 complexes (hereafter, called the original set of complexes), for which residues were found in the abstracts retrieved by the OR-queries, using three optimal SVM models (MF50L, AF138L and AF24L), trained and validated on the 1,305 abstracts retrieved by the AND-queries (see above). In this approach, an abstract is classified either as positive (publication in the PPI context) or negative (not PPI-relevant context) and then only positive abstracts are retained for the *P*_TM_ calculation (Eq 1).

**Fig 5:**
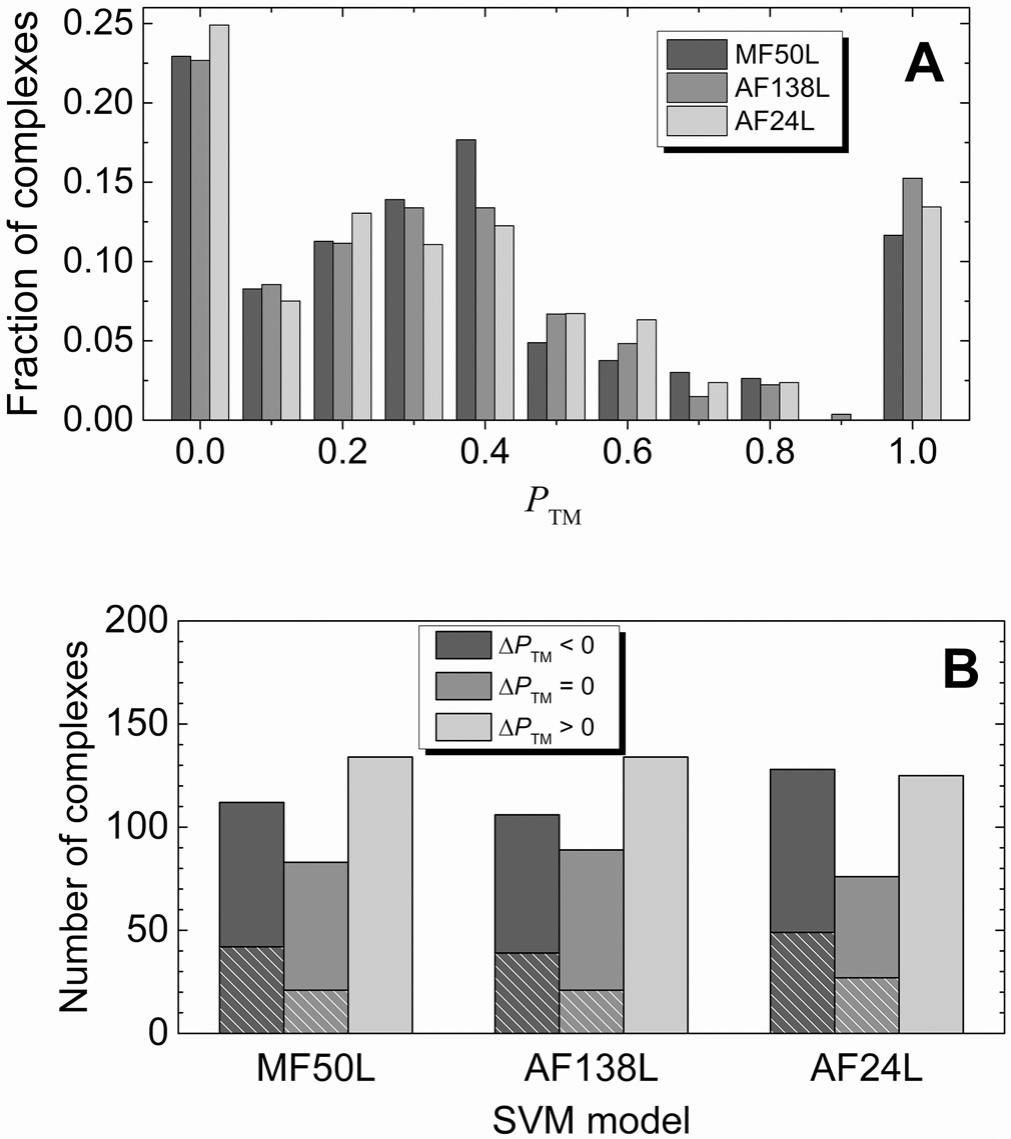
Performance of the best SVM models. The abstracts were retrieved by the OR-queries. Distribution of complexes (A) is shown according to the TM performance, *P*_TM_ (Eq 1). The distribution is normalized by the total number of complexes for which residues were identified (column 2 in Table 3). After filtering of abstracts by the optimal models, for a number of complexes (B) *P*_TM_ improves (Δ*P*_TM_ > 0), does not change (Δ*P*_TM_ = 0) and gets worse (Δ*P*_TM_ < 0). Hatched areas show the number of complexes, for which the optimal models removed all abstracts.

MF50L, AF138L and AF24L models removed at least one abstract for 296, 294 and 302 complexes, respectively, which is ~ 90% of the initial set. Overall performance of the SVM-enhanced TM did not change significantly (middle part of Table 3 and Fig 5A) compared to the basic TM (upper part of Table 3 and Fig 2). However, complexes, for which SVM models erroneously remove interface residues (*P*_TM_ decreases) constitute only ~ 1/3 of the initial set (Fig 5B). The SVM models filtered out all PPI-relevant and all PPI-irrelevant (with non-interface residues) abstracts for ~13% and ~ 7% of the initial dataset, respectively (hatched parts of the Δ*P*_TM_ < 0 and Δ*P*_TM_ = 0 bars in Fig 5B).

Analysis of performance of the SVM models on several complexes (S1 Table and S2 Text) revealed somewhat erratic performance of different models, caused by the inconsistency in residue context where interface residues are present in the abstracts with prevailing non-PPI features and vice versa (e.g., Ala11 of TIMP3 was found in the abstract of study about vasoconstricting peptide administration, not directly relevant to this protein binding [52]).

### Docking with the text mining constraints

We ran the free docking by GRAMM to model complexes of unbound proteins from the Dockground X-ray benchmark 3 [46] using constraints generated by the basic TM protocol with OR-queries (see Methods).

In the unbound set of 99 complexes, by design the component proteins have both the co-crystallized and separately resolved X-structures, and as such were presumably on average more extensively studied than the complexes from the main bound set of 579 complexes used in this study for TM evaluation. This resulted in a significantly larger pool of publications extracted by the OR-queries (68 abstracts per complex for the unbound set, compared to 32 abstracts per complex for the bound set). Thus, a significantly larger number of residues per complex were identified (S13 Fig) and the TM performed better on the unbound set than on the larger bound one (last row in Table 3). However, the number of irrelevant (non-interface) residues was also significantly larger, reducing TM effectiveness (S14 Fig). The AND-queries retrieved abstracts with residues for 37 complexes only and TM protocol with the AND-queries was not used here separately. However, for residue ranking (Eq 7), we kept track of which residues were retrieved by the AND-queries. The TM results based on OR-queries for the top 10 residues per complex (5 for each protein, ranked by the frequency of the residue occurrence, Eq 7) were significantly better (S14 Fig). Thus, these residues were submitted to GRAMM docking program for scoring of the docking scan output.

To single out the role of the TM constraints, they were applied to re-rank unrefined and otherwise unscored docking models output directly from the GRAMM scan (the baseline for evaluating the impact of the TM constraints). For comparison, the re-ranking was also done separately with the correct interface residues as constraints (see Methods). We used strict (at least, one model with *i*-RMSD ≤ 5 Å in top 10 predictions) and relaxed (at least, one model with *i*-RMSD ≤ 8 Å in top 100 predictions) success criteria.

**Fig 6:**
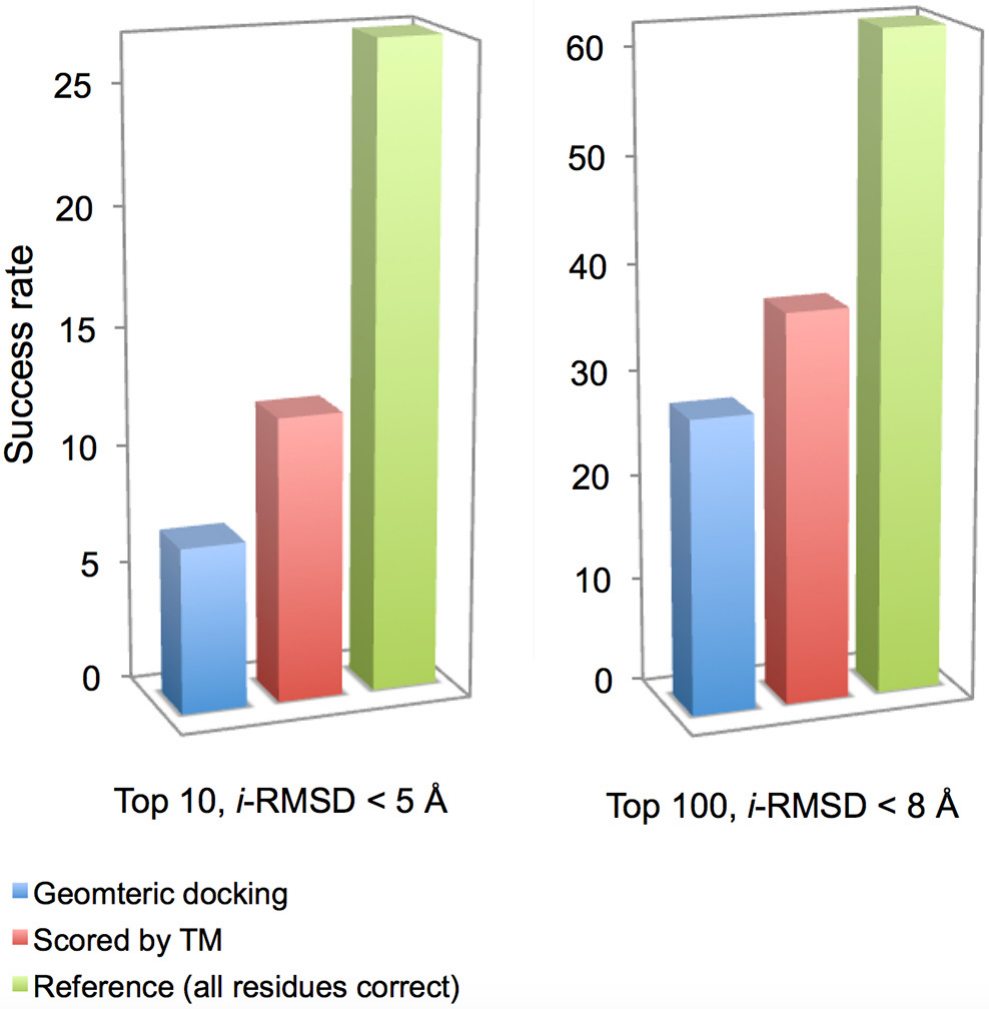
Docking with TM constraints. The results of benchmarking on the unbound X-ray set from Dockground. A complex was predicted successfully if at least one in top ten matches had ligand C^α^ interface RMSD ≤ 5 Å (A), and one in top hundred had RMSD ≤ 8 Å (B). The success rate is the percentage of successfully predicted complexes in the set. The low-resolution geometric scan output (20,000 matches) from GRAMM docking, with no post-processing, except removal of redundant matches, was scored by TM results. The reference bars show scoring by the actual interface residues (see text).

The TM scoring significantly increased docking success rates, by 71% (compared to the baseline shown as blue columns in Fig 6) according to the stricter criterion, and by 32% according to the relaxed one (Fig 6). The results on the reference set of constraints, corresponding to the correct interface residues, showed that 27 complexes have near-native matches in the top 20,000 scan predictions according to the strict criterion, and 62 according to the relaxed one. The RMSD was calculated between the unbound ligand predicted match and the unbound ligand structurally aligned with the bound in the complex. Such alignment has significant mismatches with the receptor in a number of complexes, due to the conformational change upon binding. So the near-native matches for such complexes cannot be predicted by the surface complementarity-based rigid body free docking (this correlates with the docking decoys results [53] where a near-native match was found only for 61 complexes in 500,000 top scan matches).

The observed increase of the docking success rate is the result of constraints from the basic TM only. One can assume that the deep parsing/NLP will lead to further improvement of the docking quality, closer to the level of the reference constraints (Fig 6).

### Conclusions

TM has been widely used in recreating PPI networks, as well as in detecting functional sites (small ligand binding sites) on protein structures. Combining and expanding these two well-developed research areas, we applied TM to structural modeling of protein-protein complexes (protein docking). Abstracts of publications on 579 protein complexes from Dockground were retrieved from PubMed, using AND- and OR-queries (both proteins and at least one protein mentioned in the text, correspondingly). The AND-queries identified more correct residues than the OR-queries, but retrieved abstracts with residues for significantly less complexes. SVM was used to improve the performance of OR-queries. The SVM models generated using simple bag-of-words representation of the text, removed irrelevant information extracted by the OR-queries, albeit not enough for an accurate discrimination of non-interface from the interface residues, as shown by the inconsistent performance of different SVM models. Whereas human expertise can consistently distinguish relevant from non-relevant to the interface information (as shown by our evaluation of a small subset of abstracts), a reliable and accurate automated procedure requires greater sophistication than the basic one used in our study.

The basic TM was used to generate constraints for docking, and tested on the protein-protein unbound docking benchmark set. TM significantly increased the docking success rates. Contextual analysis by deep parsing on sentence/residue level (an on-going study in our group) should improve the detection of the interface residues, and further increase the docking success rates. The preliminary results in this proof-of-concept study showed that TM is a promising approach to protein docking, with its utility increasing along with the rapidly growing amount of publicly available information on protein complexes.

## Supporting Information

S1 Text Performance of basic text mining for specific protein-protein complexes. (PDF)

S2 Text Performance of SVM-enhanced text mining for specific protein-protein complexes. (PDF)

S1 Table Examples of optimal SVM model impact on TM output. (PDF)

S1 Fig Examples of residues extracted by the basic TM. (PDF)

S2 Fig Distribution of complexes according to the quality of the basic TM, accounting for mismatch between residue numbering in PDB and UniProt sequences. (PDF)

S3 Fig Distribution of complexes according to the quality of the basic TM, excluding abstracts published after the paper on the original PDB structure. (PDF)

S4 Fig SVM performance for manual feature selection using linear kernel. (PDF)

S5 Fig SVM performance for manual feature (50_NM) selection using polynomial kernel with different degrees. (PDF)

S6 Fig SVM performance for manual feature selection using RBF kernel with γ = 1. (PDF)

S7 Fig SVM performance for manual feature (20_NM) selection using RBF kernel with various γ. (PDF)

S8 Fig SVM performance for automated feature selection using linear kernel and 0.05 margin. (PDF)

S9 Fig SVM performance for automated feature selection using linear kernel without margin. (PDF)

S10 Fig SVM performance for automated feature selection using polynomial kernel with different degrees and no margin. (PDF)

S11 Fig SVM performance for automated feature selection using RBF kernel with different γ and no margin. (PDF)

S12 Fig Comparison of Matthews correlation coefficient for different approaches to calculate the number of features in abstracts in the training set. (PDF)

S13 Fig Distribution of total number of residues per complex extracted by OR-queries in two sets. (PDF)

S14 Fig Normalized distribution of complexes in the DOCKGROUND benchmark set 3 according to TM performance, PTM (Eq 1). (PDF)
