# Supplementary material for: Text Mining for Protein Docking
Source: PLoS Comput Biol. 2015 Dec 9;11(12):e1004630. doi: 10.1371/journal.pcbi.1004630 (PMC4674139; doi:10.1371/journal.pcbi.1004630)
Supplement: S1 Text — (PDF) [file pcbi.1004630.s001.pdf]

Performance of basic text mining for specific protein-protein complexes

SH2D1A – p59Fyn complex (1m27). For this complex, AND-query did not retrieve any abstracts. The OR-query identified 6 abstracts with 4 residues, out of which 3 are at the interface (Figure 3 in the main text, P_{TM} =0.75). Arg78 of SH2D1A protein (1m27, chain A) was detected in the abstract on the role of tyrosine kinase Fyn and SLAM (synonym of SH2D1A) interaction in the development of natural killer T cells in human and mice [1]. The abstract was not detected by AND-query because p59Fyn names that do not follow UniProt nomenclature. Arg78 was also pinpointed in two mutagenesis studies on the role of the SLAM-SAP-Fyn signaling pathway in mice CD4 T cell function and germinal center development [2, 3]. Trp119 of p59Fyn (1m27, chain C) was detected in the abstract on interactions of SH2 and SH3 domains of p59Fyn [4]. The p59Fyn residue Tyr132 was detected in an abstract of site-directed mutagenesis studies that prove importance of this residue in formation of phosphoprotein pp21 [5]. Non-interface SH2D1A residue Thr53 was found in the abstract of computational study on the role of SLAM mutations in manifestation of an immunodeficiency disease, X-linked lymphoproliferative syndrome [6]. Another residue mentioned in this abstract (Arg32) belongs to the protein core and thus did not pass protocol filters.

TACE – TIMP3 complex (3cki). For human tumor necrosis factor alpha converting enzyme (TACE) co-crystallized with the metalloproteinase inhibitor 3, TIMP3, the AND-query predicted 7 interface and 5 non-interface residues (Figure S1A, P_{TM} = 0.58), mentioned in the abstracts of 6 publications [7-12], other than the original X-ray study [13]. Redesign of metalloproteinase inhibitor TIMP1, using TIMP3 as a scaffold identified three residues, Ser4, Leu67, and Arg84, important for the TIMP3-TACE binding [7]. Ser4 and Thr2 were found to be functionally important by measuring binding affinities of the mutated TIMP3 [8]. Redesign of metalloproteinase inhibitor TIMP2, using TIMP3 and TIMP1 as scaffolds [9], identified Ser4 and, additionally, Phe34 residues. Three other residues mentioned in that abstract (Val/Leu69, Thr/Leu98 and Leu/Ile/Met100) did not match residues in the original PDB (Gly69, Val98, and Arg100, respectively). Phe34 was also mentioned in the original X-ray crystallography paper [13]. In the study of TACE cysteine-rich domains role in the TIMP-3 inhibitory potency [10], Lys315 was mentioned as a residue adjacent to the TACE catalytic site, which interacts with TIMP-3 Glu31, which is close to, but not at the TACE-TIMP3 interface. In this abstract, other non-interface residues, Lys26, Lys27, Lys30, and Lys76 were also mentioned along with residues (Glu26, Glu27, Glu30, and Glu76) that either did not match PDB numbering or did not follow residue patterns considered in this study (Table 1). Finally, Pro5 is identified in the analysis of expression levels of different human prostatic tumor cell lines [11]. However, Pro5 in this publication stands for the name of cell line rather than a residue. Ser4 was also identified by another publication [9] where the binding affinity of the mutant is better than TIMP-3. All the above residues belong to TIMP3 chain in the original PDB, except Lys315, which belongs to the TACE chain.

The OR-query for this complex found three additional abstracts [14-16] with two additional interface residues (TACE Val353 and TIMP3 Ala11) and one non-interface (TIMP3 Gly119) residue (P_{TM} = 0.60). Val353 was identified as functionally important in the study on stabilization of the TACE autoproteolysis [14]. Ala11 is mentioned in the abstract of paper [15] showing that activation of brain ET(B) receptors causes TIMP-1 and TIMP-3 production. Gly119 is present in the abstract of study [16] on mutated

growth hormone (bGH) in transgenic mice. This abstract was picked due to the presence of the TIMP-3 name, however Gly119 mentioned therein belongs to bGH protein and just accidentally coincides with the Gly119 residue in the original PDB file. The OR-query also found three additional abstracts with five residues already picked up by the AND-query. Study [17] determined that Lys26, Lys27, Lys30, and Lys76 constitute another TIMP3 binding site with the extracellular matrix (ECM). Other residues mentioned in that abstract (Arg163 and Lys165) belong to the C-terminal of TIMP3 not present in the original PDB file. Lys27 was also present in the abstract of study [18] suggesting that EZH2 (Enhancer of zeste homolog 2) accelerates lung cancer cell migration partially through repression of TIMP-3 expression. Thr2 was identified in the paper [19] showing that mutated N-TIMP3 inhibits degradation of ADAMTS-4 and ADAMTS-5 metalloproteinases.

Complex of Plectin-1 and Integrin beta-4 (3f7p). For this complex, AND-query found 3 abstracts with 4 interface residues of Integrin beta 4 (Figure S1B, P_{TM} = 1.00). Abstract in Ref. [20] states that mutations at Arg1225 and Arg1281 sites inhibit interaction of the integrin beta 4 with plectin while mutation of Lys1279 has no effect to recruit plectin. In addition, Arg1281 was spotted in the abstract in Ref. [21] also showing that mutation at this site affects the interaction between the two proteins. Another mutagenesis study [22] shows that residue Ser1325 is important for recruitment of plectin into hemidesmosomes *in vivo*.

The OR-query for this complex identified one additional abstract with one additional non-interface residue (Figure S1B) thus reducing TM performance to P_{TM} = 0.80. Arg239 was found in the abstract in Ref. [23] on interaction between plectin and glial fibrillary acidic protein (GFAP). However, the number of this residue belonging to GFAP protein, just accidentally coincides with the number of one of the arginine residues in the original PDB file. The OR-query has also retrieved 2 additional abstracts of mutagenesis studies containing Arg1281 pinpointed by the AND-query. Mutation at Arg1281 was found to effect severity of epidermolysis bullosa (genetic skin disease) [24] and to inhibit interaction between plectin and integrin beta 4 with its alpha 6 chain [25].

IRF3 – CBP complex (1zoq). For this complex, the AND-query identified 6 abstracts with 3 non-interface residues in the Interferon regulatory factor 3, IRF3 (Figure S1C, P_{TM} = 0.00). All found residues (Ser339 [26, 27], Ser385 [28-30], and Ser386 [28-31]) were studied in the context of their phosphorylation, which regulates CBP binding allosterically. Interferon interacts with many partners (e.g., BioGrid database [32] lists 44 interactions for IRF-3) and phosphorylation of these three residues was studied in the context of IRF3 binding to other proteins as well. The OR-query thus found Ser339, Ser385 and Ser386 residues in one [33], two [34, 35], and five [35-39] additional abstracts, respectively.

The OR-query for this complex found one more residue, Leu238 of IRF3, in one additional abstract (P_{TM} = 0.00) of study on the interferon (IFN) role in resisting evasion of the African swine fever virus into pig immune system [40].

References

1. Nunez-Cruz S, Yeo WC, Rothman J, Ojha P, Bassiri H, et al. Differential requirement for the SAP-Fyn interaction during NK T cell development and function. J Immunol. 2008;181:2311-20.

2. McCausland MM, Yusuf I, Tran H, Ono N, Yanagi Y, et al. SAP regulation of follicular helper CD4 T cell development and humoral immunity is independent of SLAM and Fyn kinase. J Immunol. 2007;178:817-28.

3. Cannons JL, Yu LJ, Jankovic D, Crotty S, Horai R, et al. SAP regulates T cell-mediated help for humoral immunity by a mechanism distinct from cytokine regulation. J Exp Med. 2006;203:1551-65.

4. Panchamoorthy G, Fukazawa T, Stolz L, Payne G, Reedquist K, et al. Physical and functional interactions between SH2 and SH3 domains of the Src family protein tyrosine kinase p59fyn. Mol Cell Biol. 1994;14:6372-85.

5. Koyasu S, McConkey DJ, Clayton LK, Abraham S, Yandava B, et al. Phosphorylation of multiple CD3 zeta tyrosine residues leads to formation of pp21 in vitro and in vivo. Structural changes upon T cell receptor stimulation. J Biol Chem. 1992;267:3375-81.

6. Chandrasekaran P, Rajasekaran R. Structural characterization of disease-causing mutations on SAP and the functional impact on the SLAM peptide: A molecular dynamics approach. Mol Biosyst. 2014;10:1869-80.

7. Lee MH, Maskos K, Knauper V, Dodds P, Murphy G. Mapping and characterization of the functional epitopes of tissue inhibitor of metalloproteinases (TIMP)-3 using TIMP-1 as the scaffold: A new frontier in TIMP engineering. Protein Sci. 2002;11:2493-503.

8. Lee MH, Verma V, Maskos K, Nath D, Knauper V, et al. Engineering N-terminal domain of tissue inhibitor of metalloproteinase (TIMP)-3 to be a better inhibitor against tumour necrosis factor-α-converting enzyme. Biochem J. 2002;364:227-34.

9. Lee MH, Rapti M, Murphy G. Delineating the molecular basis of the inactivity of tissue inhibitor of metalloproteinase-2 against tumor necrosis factor-α-converting enzyme. J Biol Chem. 2004;279:45121-9.

10. Lee MH, Dodds P, Verma V, Maskos K, Knauper V, et al. Tailoring tissue inhibitor of metalloproteinases-3 to overcome the weakening effects of the cysteine-rich domains of tumour necrosis factor-alpha converting enzyme. Biochem J. 2003;371:369-76.

11. Karan D, Lin FC, Bryan M, Ringel J, Moniaux N, et al. Expression of ADAMs (a disintegrin and metalloproteases) and TIMP-3 (tissue inhibitor of metalloproteinase-3) in human prostatic adenocarcinomas. Int J Oncol. 2003;23:1365-71.

12. Lee MH, Rapti M, Knauper V, Murphy G. Threonine 98, the pivotal residue of tissue inhibitor of metalloproteinases (TIMP)-1 in metalloproteinase recognition. J Biol Chem. 2004;279:17562-9.

13. Wisniewska M, Goettig P, Maskos K, Belouski E, Winters D, et al. Structural determinants of the ADAM inhibition by TIMP-3: crystal structure of the TACE-N-TIMP-3 complex. J Mol Biol. 2008;381:1307-19.

14. Ingram RN, Orth P, Strickland CL, Le HV, Madison V, et al. Stabilization of the autoproteolysis of TNF-alpha converting enzyme (TACE) results in a novel crystal form suitable for structure-based drug design studies. Protein Eng. 2006;19:155-61.

15. Koyama Y, Baba A, Matsuda T. Intracerebroventricular administration of an endothelin ETB receptor agonist increases expression of tissue inhibitor of matrix metalloproteinase-1 and -3 in rat brain. Neuroscience. 2007;147:620-30.

16. Esposito C, Liu ZH, Striker GE, Phillips C, Chen NY, et al. Inhibition of diabetic nephropathy by a GH antagonist: a molecular analysis. Kidney Int. 1996;50:506-14.

17. Lee MH, Atkinson S, Murphy G. Identification of the extracellular matrix (ECM) binding motifs of tissue inhibitor of metalloproteinases (TIMP)-3 and effective transfer to TIMP-1. J Biol Chem. 2007;282:6887-98.

18. Xu C, Hou Z, Zhan P, Zhao W, Chang C, et al. EZH2 regulates cancer cell migration through repressing TIMP-3 in non-small cell lung cancer. Med Oncol. 2013;30:1-8.

19. Lim N, Kashiwagi M, Visse R, Jones J, Enghild J, et al. Reactive-site mutants of N-TIMP-3 that selectively inhibit ADAMTS-4 and ADAMTS-5: Biological and structural implications. Biochem J. 2010;431:113-22.

20. Koster J, Kuikman I, Kreft M, Sonnenberg A. Two different mutations in the cytoplasmic domain of the integrin beta 4 subunit in nonlethal forms of epidermolysis bullosa prevent interaction of beta 4 with plectin. J Invest Dermatol. 2001;117:1405-11.

21. Nievers MG, Kuikman I, Geerts D, Leigh IM, Sonnenberg A. Formation of hemidesmosome-like structures in the absence of ligand binding by the (alpha)6(beta)4 integrin requires binding of HD1/plectin to the cytoplasmic domain of the (beta)4 integrin subunit. J Cell Sci. 2000;113 (Pt 6):963-73.

22. Litjens SH, Wilhelmsen K, de Pereda JM, Perrakis A, Sonnenberg A. Modeling and experimental validation of the binary complex of the plectin actin-binding domain and the first pair of fibronectin type III (FNIII) domains of the beta4 integrin. J Biol Chem. 2005;280:22270-7.

23. Tian R, Gregor M, Wiche G, Goldman JE. Plectin regulates the organization of glial fibrillary acidic protein in Alexander disease. Am J Pathol. 2006;168:888-97.

24. Pulkkinen L, Rouan F, Bruckner-Tuderman L, Wallerstein R, Garzon M, Brown T, et al. Novel ITGB4 mutations in lethal and nonlethal variants of epidermolysis bullosa with pyloric atresia: missense versus nonsense. Am J Hum Genet. 1998;63:1376-87.

25. Kambham N, Tanji N, Seigle RL, Markowitz GS, Pulkkinen L, Uitto J, et al. Congenital focal segmental glomerulosclerosis associated with beta4 integrin mutation and epidermolysis bullosa. Am J Kidney Dis. 2000;36:190-6.

26. Wang JT, Doong SL, Teng SC, Lee CP, Tsai CH, et al. Epstein-Barr virus BGLF4 kinase suppresses the interferon regulatory factor 3 signaling pathway. J Virol. 2009;83:1856-69.

27. Clement JF, Bibeau-Poirier A, Gravel SP, Grandvaux N, Bonneil E, et al. Phosphorylation of IRF-3 on Ser 339 generates a hyperactive form of IRF-3 through regulation of dimerization and CBP association. J Virol. 2008;82:3984-96.

28. Chen W, Srinath H, Lam SS, Schiffer CA, Royer WE, et al. Contribution of Ser386 and Ser396 to activation of interferon regulatory factor 3. J Mol Biol. 2008;379:251-60.

29. Panne D, McWhirter SM, Maniatis T, Harrison SC. Interferon regulatory factor 3 is regulated by a dual phosphorylation-dependent switch. J Biol Chem. 2007;282:22816-22.

30. Mori M, Yoneyama M, Ito T, Takahashi K, Inagaki F, et al. Identification of Ser-386 of interferon regulatory factor 3 as critical target for inducible phosphorylation that determines activation. J Biol Chem. 2004;279:9698-702.

31. Takahasi K, Horiuchi M, Fujii K, Nakamura S, Noda NN, et al. Ser386 phosphorylation of transcription factor IRF-3 induces dimerization and association with CBP/p300 without overall conformational change. Genes Cells. 2010;15:901-10.

32. Chatr-Aryamontri A, Breitkreutz BJ, Oughtred R, Boucher L, Heinicke S, et al. The BioGRID interaction database: 2015 update. Nucl Acid Res. 2015;43:D470-8.

33. Saitoh T, Tun-Kyi A, Ryo A, Yamamoto M, Finn G, al. e. Negative regulation of interferon-regulatory factor 3-dependent innate antiviral response by the prolyl isomerase Pin1. Nat Immunol. 2006;7:598-605.

34. Gu M, Zhang T, lin W, Liu Z, Lai R, et al. Protein phosphatase PP1 negatively regulates the Toll-like receptor- and RIG-I-like receptor-triggered production of type I interferon by inhibiting IRF3 phosphorylation at serines 396 and 385 in macrophage. Cell Signal. 2014;26:2930-9.

35. Bergstroem B, Johnsen IB, Nguyen TT, Hagen L, Slupphaug G, et al. Identification of a novel in vivo virus-targeted phosphorylation site in interferon regulatory factor-3 (IRF3). J Biol Chem. 2010;285:24904-14.

36. Anglero-Rodriguez YI, Pantoja P, Sariol CA. Dengue virus subverts the interferon induction pathway via NS2B/3 protease-IkappaB kinase epsilon interaction. Clin Vaccine Immunol. 2014;21:29-38.

37. Shu C, Sankaran B, Chaton CT, Herr AB, Mishra A, et al. Structural insights into the functions of TBK1 in innate antimicrobial immunity. Structure. 2013;21:1137-48.

38. Inoue K, Tsukiyama-Kohara K, Matsuda C, Yoneyama M, Fujita T, et al. Impairment of interferon regulatory factor-3 activation by hepatitis C virus core protein basic amino acid region 1. Bioch Biophys Res Comm. 2012;428:494-9.

39. Fujii K, Nakamura S, Takahashi K, Inagaki F. Systematic characterization by mass spectrometric analysis of phosphorylation sites in IRF-3 regulatory domain activated by IKK-i. J Proteomics. 2010;73:1196-203.

40. Correia S, Ventura S, Parkhouse RM. Identification and utility of innate immune system evasion mechanisms of ASFV. Virus Res. 2013;173:87-100.
