# Supplementary material for: Text Mining for Protein Docking
Source: PLoS Comput Biol. 2015 Dec 9;11(12):e1004630. doi: 10.1371/journal.pcbi.1004630 (PMC4674139; doi:10.1371/journal.pcbi.1004630)
Supplement: S2 Text — (PDF) [file pcbi.1004630.s002.pdf]

Performance of SVM-enhanced text mining for specific protein-protein complexes

For the TACE-TIMP3 complex (3cki, Figure S1A), all three models removed non-interface Gly119 and one or both interface residues brought up by the OR-query (Table S1). In addition, all models removed one interface residue from the AND-query abstracts (Pro5B) and overlooked all non-interface residues found by the AND-query. This led to slightly deteriorated TM performance (P_{TM} drops by 0.02 for the MF50L and AF138L models and by 0.06 for AF24L model). For the Plectin 1 and Integrin beta 4 complex (3f7p, Figure S1B), the AF138L and AF24L models removed plectin non-interface Arg239, detected by the OR-query, thus increasing P_{TM} to 1.00. The MF50L model, however, also removed all interface residues detected by the AND-query, thereby erroneously excluding this complex from the consideration (Table S1). Still, for the SH2D1A-p59Fyn complex (1m27, Figure 3), the MF50L model raised P_{TM} to 1.00 but, in contrast, the AF138L and AF24L models performed worse, dropping P_{TM} to 0.67 (Table S1). These models purged p59Fyn interface residue Tyr132. Finally, the AF138L and AF24L models correctly excluded IRF3-CBP complex (1zoq, Figure S1C) from consideration removing all non-interface residues picked up by the OR-query while the MF50L model succeeded only partially, removing only one non-interface residue (Table S1). This model overlooked other non-interface residues.
