# Supplementary material for: Text Mining for Protein Docking
Source: PLoS Comput Biol. 2015 Dec 9;11(12):e1004630. doi: 10.1371/journal.pcbi.1004630 (PMC4674139; doi:10.1371/journal.pcbi.1004630)
Supplement: S1 Table — (PDF) [file pcbi.1004630.s003.pdf]

Table S1. *Examples of optimal SVM model impact on TM output.* The examples are from Figures 3 and S1. A residue is considered removed if an SVM model filtered out all abstracts mentioning this residue.

**SVM model**	**PDB 3cki**		**PDB 3f7p**		**PDB 1m27**		**PDB 1zoq**	
	**CR** [a]	**IR** [b]	**CR**	**IR**	**CR**	**IR**	**CR**	**IR**
**MF50L**	Gly119B	Pro5B Ala11B	Arg239A	Arg1225C Lys1279C Arg1281C Ser1325C	Thr53A	Tyr132C Trp119C	Ser339A	–
**AF138L**	Gly119B	Pro5B Val353A	Arg239A	–	–	Tyr132C	Ser339A, Ser385A, Ser386A, Leu238A	–
**AF24L**	Gly119B	Pro5B Ala11B Val353A	Arg239A	–	–	Tyr132C	Ser339A, Ser385A, Ser386A, Leu238A	–

[a] Correctly removed (non-interface residues)
[b] Incorrectly removed (interface residues)
