# Supplementary material for: Text Mining for Protein Docking
Source: PLoS Comput Biol. 2015 Dec 9;11(12):e1004630. doi: 10.1371/journal.pcbi.1004630 (PMC4674139; doi:10.1371/journal.pcbi.1004630)
Supplement: S1 Fig — (PDF) [file pcbi.1004630.s004.pdf]

A

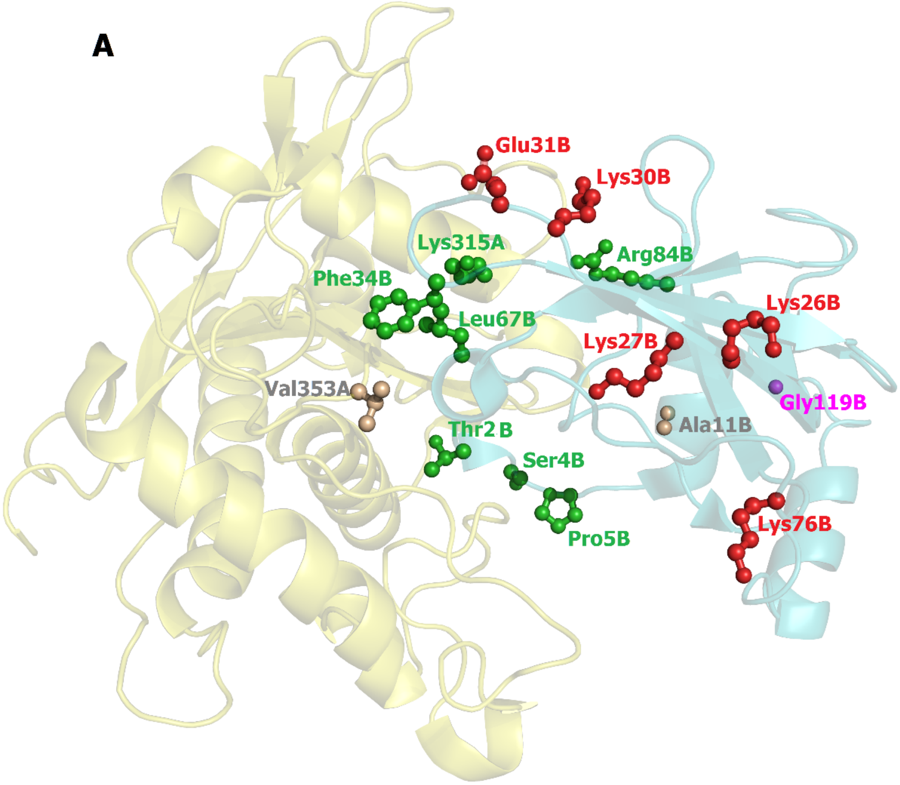
Glu31B
Lys30B
Lys315A
Arg84B
Phe34B
Leu67B
Lys26B
Lys27B
Val353A
Gly119B
Ala11B
Thr2 B
Ser4B
Pro5B
Lys76B

B

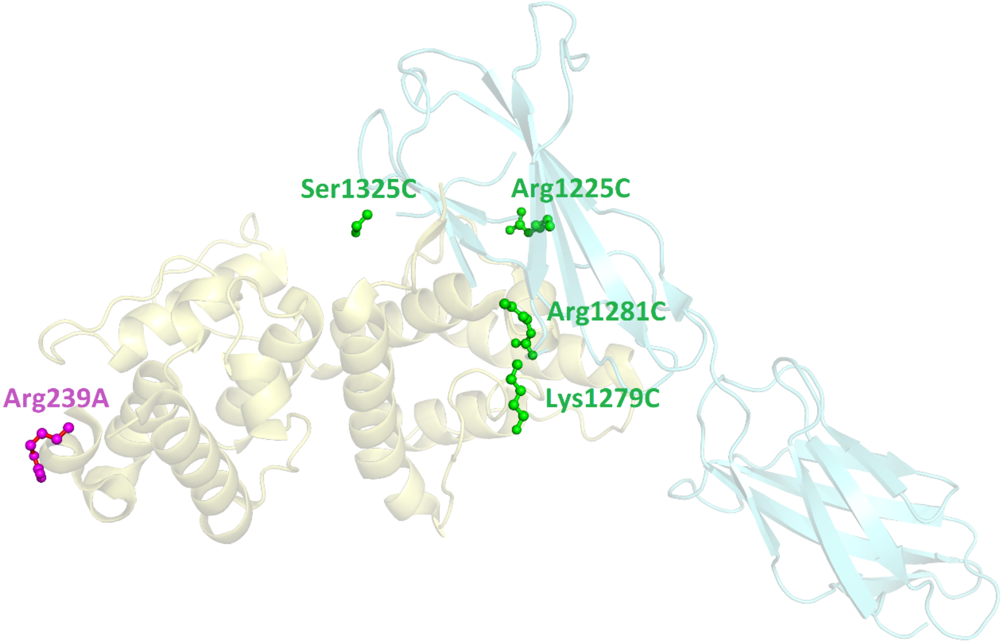
Ser1325C
Arg1225C
Arg1281C
Arg239A
Lys1279C

C

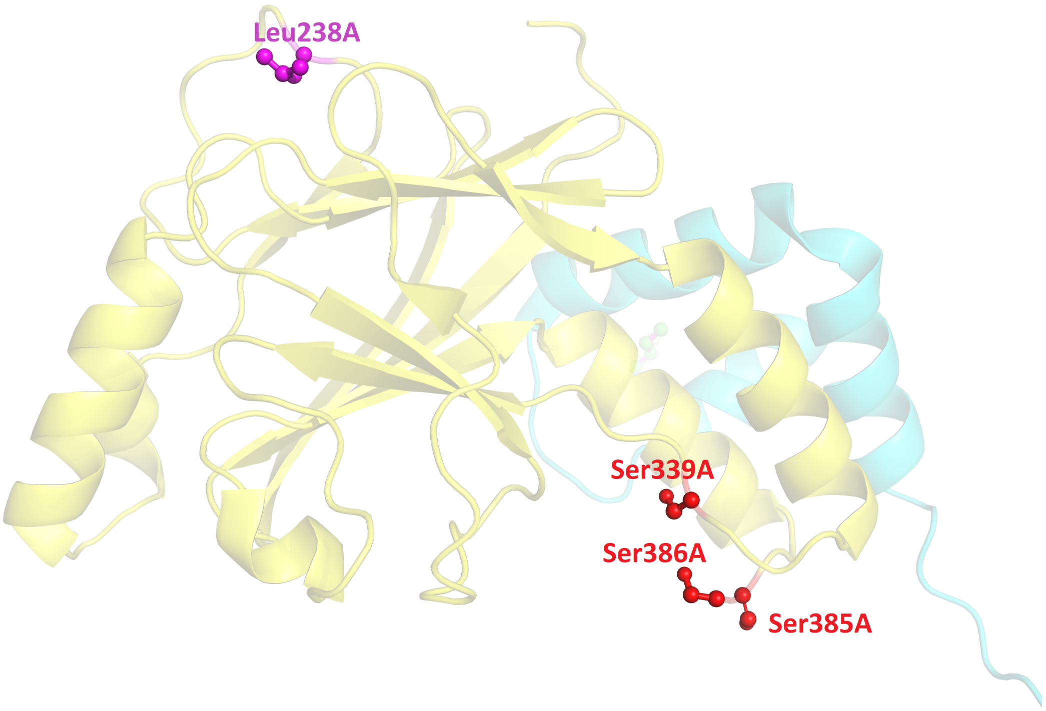

Figure S1. Examples of residues extracted by the basic TM. The structure, chain ID, and residue numbers are from 3cki (A), 3f7p (B), and 1zoq (C). Interface and non-interface residues detected by the AND-query are in green and red, respectively. Additional interface and non-interface residues detected by the OR-query are in brown and magenta, respectively.
