# Supplementary material for: Text Mining for Protein Docking
Source: PLoS Comput Biol. 2015 Dec 9;11(12):e1004630. doi: 10.1371/journal.pcbi.1004630 (PMC4674139; doi:10.1371/journal.pcbi.1004630)
Supplement: S3 Fig — (PDF) [file pcbi.1004630.s006.pdf]

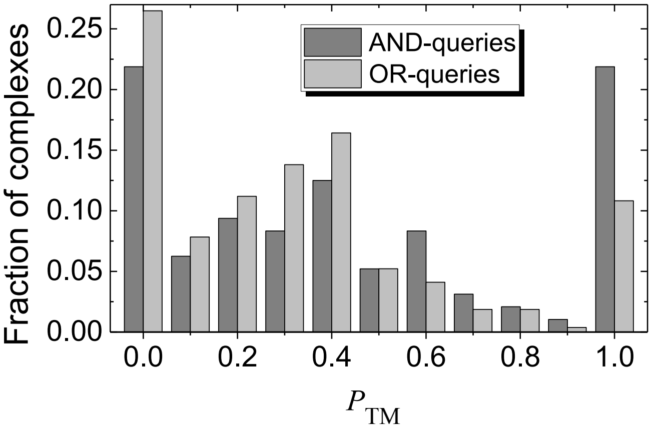

Figure S3. Distribution of complexes according to the quality of the basic TM, excluding abstracts published after the paper on the original PDB structure. The TM performance is according to P_{TM} (Eq. 1). The distribution is normalized to the total number of complexes for which residues were identified (column 3 in Table 3).
