# Supplementary material for: Text Mining for Protein Docking
Source: PLoS Comput Biol. 2015 Dec 9;11(12):e1004630. doi: 10.1371/journal.pcbi.1004630 (PMC4674139; doi:10.1371/journal.pcbi.1004630)
Supplement: S4 Fig — (PDF) [file pcbi.1004630.s007.pdf]

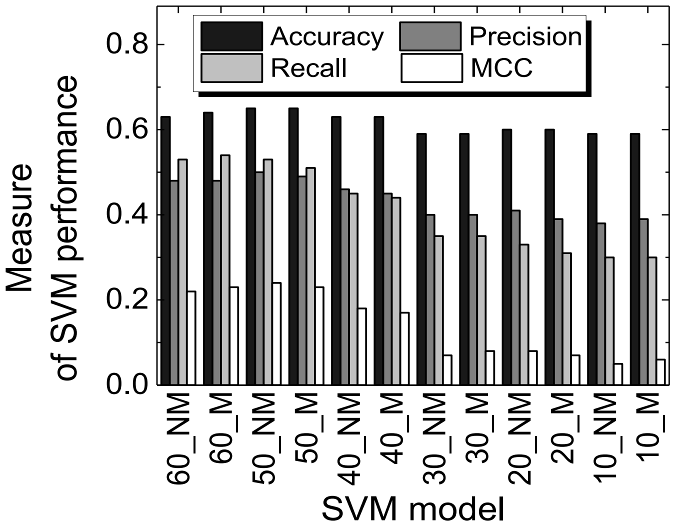

Figure S4. SVM performance for manual feature selection using linear kernel. Data with M suffix was obtained on abstracts excluding those with SVM-scores -0.05 to +0.05. Data with NM suffix was obtained on all abstracts.
