# Supplementary material for: Text Mining for Protein Docking
Source: PLoS Comput Biol. 2015 Dec 9;11(12):e1004630. doi: 10.1371/journal.pcbi.1004630 (PMC4674139; doi:10.1371/journal.pcbi.1004630)
Supplement: S5 Fig — (PDF) [file pcbi.1004630.s008.pdf]

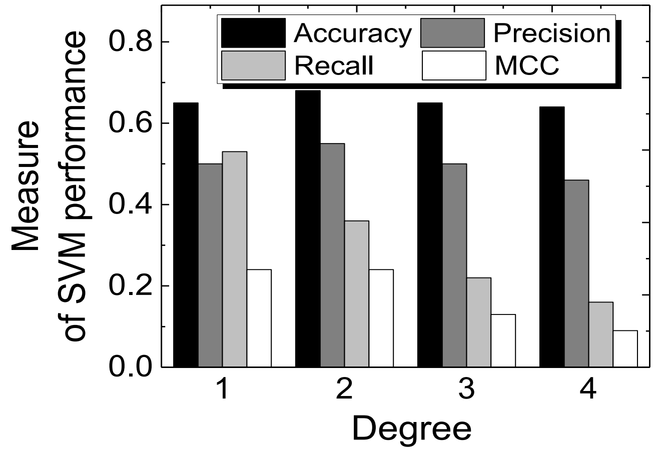

Figure S5. SVM performance for manual feature (50_NM) selection using polynomial kernel with different degrees.
