# Supplementary figures and images for: Text Mining for Protein Docking
Source: PLoS Comput Biol. 2015 Dec 9;11(12):e1004630. doi: 10.1371/journal.pcbi.1004630 (PMC4674139; doi:10.1371/journal.pcbi.1004630)

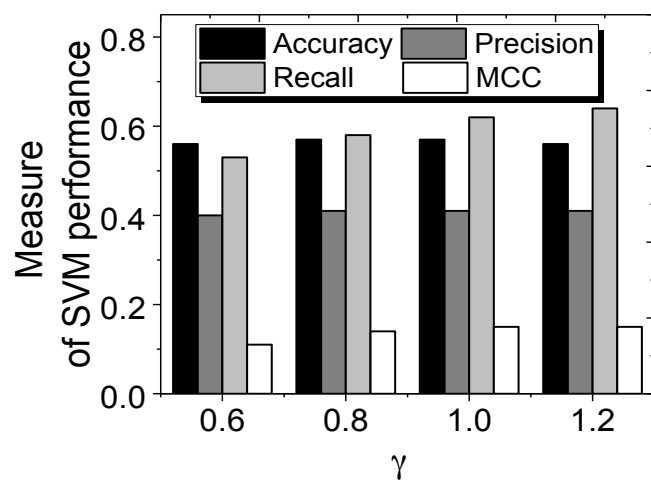

**Figure S7. SVM performance for manual feature (20\_NM) selection using RBF kernel with various  $\gamma$ .**

Supplement: S7 Fig — (PDF) [file pcbi.1004630.s010.pdf]

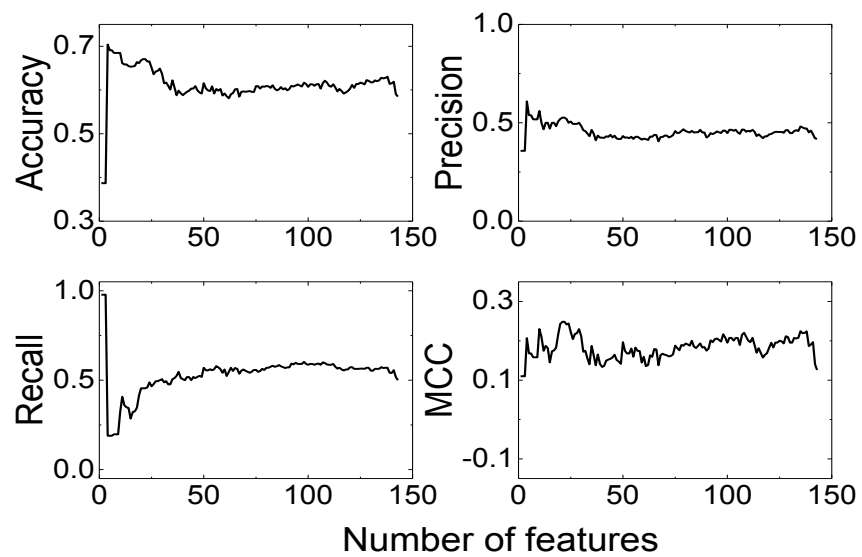

**Figure S8. SVM performance for automated feature selection using linear kernel and 0.05 margin.**

Supplement: S8 Fig — (PDF) [file pcbi.1004630.s011.pdf]

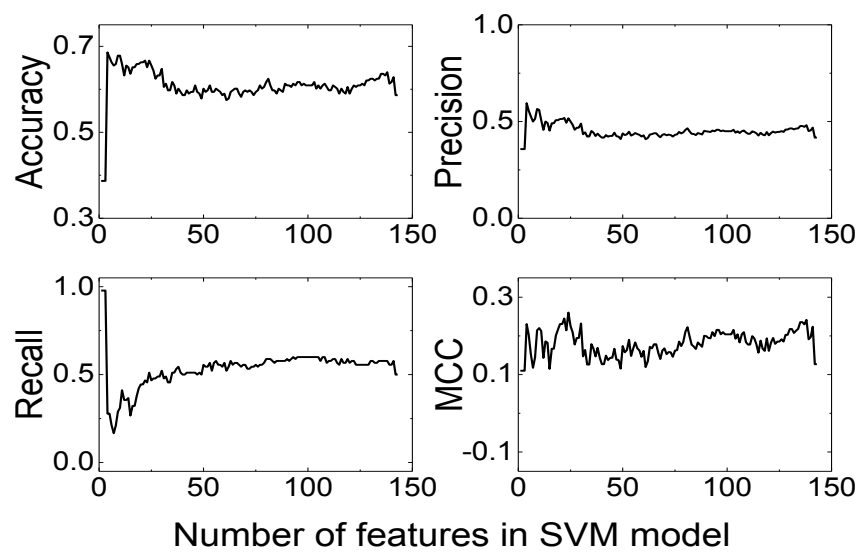

**Figure S9. SVM performance for automated feature selection using linear kernel without margin.**

Supplement: S9 Fig — (PDF) [file pcbi.1004630.s012.pdf]
