# Supplementary material for: Text Mining for Protein Docking
Source: PLoS Comput Biol. 2015 Dec 9;11(12):e1004630. doi: 10.1371/journal.pcbi.1004630 (PMC4674139; doi:10.1371/journal.pcbi.1004630)
Supplement: S10 Fig — (PDF) [file pcbi.1004630.s013.pdf]

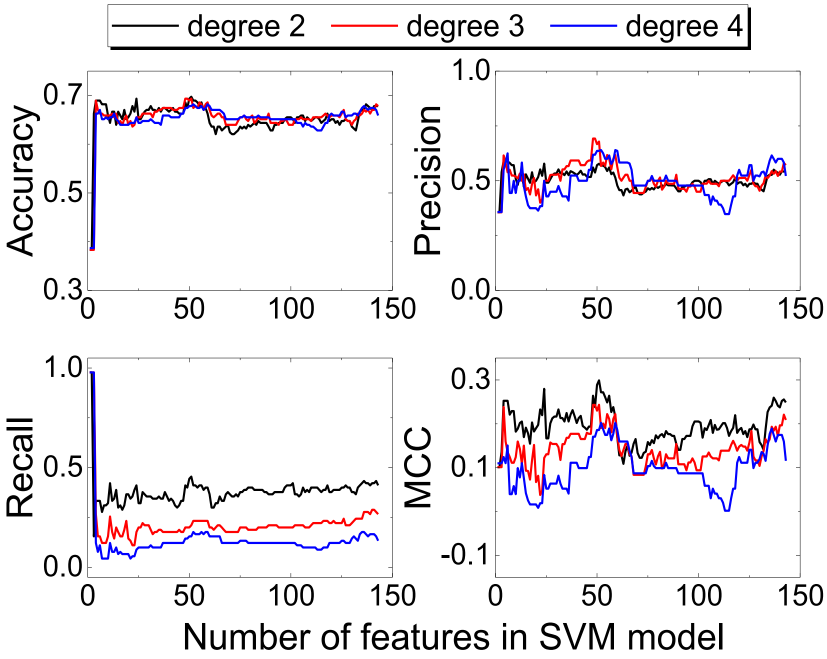

Figure S10. SVM performance for automated feature selection using polynomial kernel with different degrees and no margin.
