# Supplementary material for: Text Mining for Protein Docking
Source: PLoS Comput Biol. 2015 Dec 9;11(12):e1004630. doi: 10.1371/journal.pcbi.1004630 (PMC4674139; doi:10.1371/journal.pcbi.1004630)
Supplement: S12 Fig — (PDF) [file pcbi.1004630.s015.pdf]

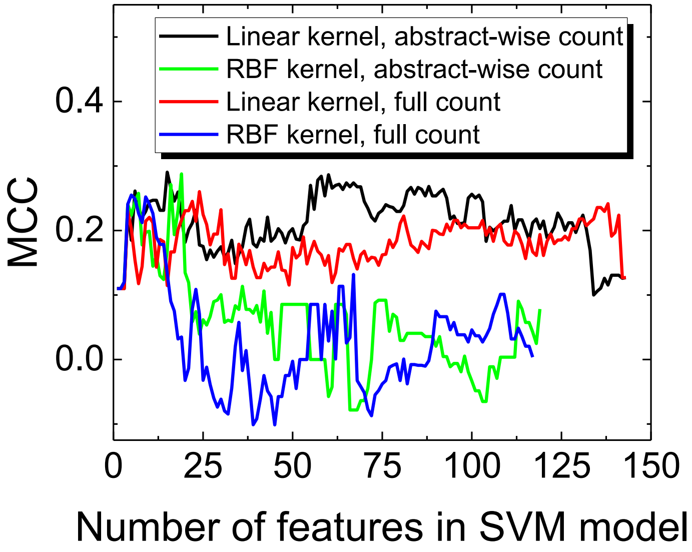

Figure S12. **Comparison of Matthews correlation coefficient for different approaches to calculate the number of features in abstracts in the training set.** The abstract-wise feature count (irrespective of the number of times the feature appears in an abstract) was used earlier in the extraction of features for prediction of protein function and localization (Wong & Shatkay, BMC Bioinformatics, 2013,14,Suppl.3: S14; Shatkay et al. Methods, 2015,74:54-64). The full count, used in the current study, accounts for all instances of a feature in an abstract. The data was obtained on the validation set of 261 abstracts. The SVM models were trained on 1,044 abstracts (see Methods).
