# Supplementary material for: Text Mining for Protein Docking
Source: PLoS Comput Biol. 2015 Dec 9;11(12):e1004630. doi: 10.1371/journal.pcbi.1004630 (PMC4674139; doi:10.1371/journal.pcbi.1004630)
Supplement: S13 Fig — (PDF) [file pcbi.1004630.s016.pdf]

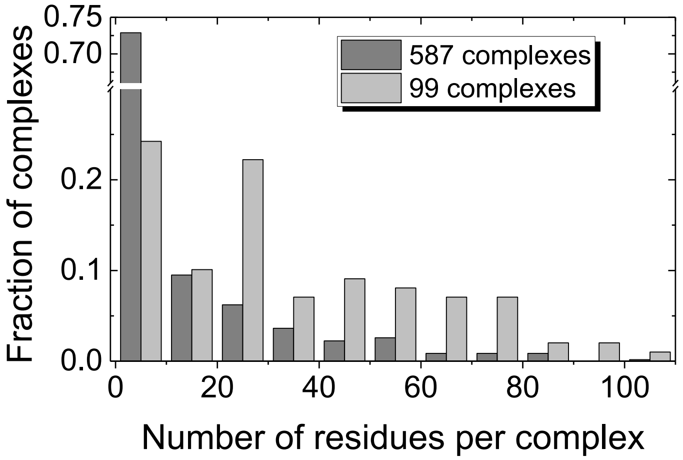

Figure S13. **Distribution of total number of residues per complex extracted by OR-queries in two sets.** The data is normalized by the total number of complexes in the corresponding set.
