# Supplementary material for: Text Mining for Protein Docking
Source: PLoS Comput Biol. 2015 Dec 9;11(12):e1004630. doi: 10.1371/journal.pcbi.1004630 (PMC4674139; doi:10.1371/journal.pcbi.1004630)
Supplement: S14 Fig — (PDF) [file pcbi.1004630.s017.pdf]

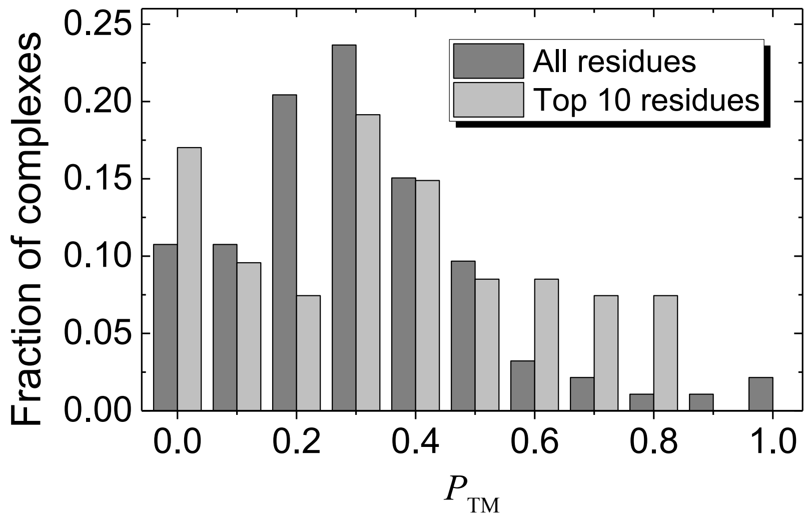

Figure S14. Normalized distribution of complexes in the DOCKGROUND benchmark set 3 according to TM performance, P_{TM} (Eq. 1). The data is obtained by the basic TM protocol with OR-queries, and is normalized to the total number of complexes, for which residues were predicted (column 3 in Table 3). Dark and light bars show distributions for all retrieved residues and for the top 10 residues, submitted to docking, respectively.
